# A novel facile synthesis of metal nitride@metal oxide (BN/Gd_2_O_3_) nanocomposite and their antibacterial and anticancer activities

**DOI:** 10.1038/s41598-023-49895-4

**Published:** 2023-12-20

**Authors:** Mayyadah H. Mohsin, Khawla S. Khashan, Ghassan M. Sulaiman, Hamdoon A. Mohammed, Kamal A. Qureshi, Ashok Aspatwar

**Affiliations:** 1 Department of Applied Sciences, University of Technology, Baghdad, Iraq; 2 Department of Medicinal Chemistry and Pharmacognosy, College of Pharmacy, Qassim University, 51452 Qassim, Saudi Arabia; 3 Department of Pharmacognosy and Medicinal Plants, Faculty of Pharmacy, Al-Azhar University, Cairo, 11884 Egypt; 4 Faculty of Medicine and Health Technology, Tampere University, 33520 Tampere, Finland

**Keywords:** Biophysics, Biotechnology, Microbiology, Nanoscience and technology

## Abstract

In this study, a novel core/shell nanocomposite structure (*h*-BN@Gd_2_O_3_ NCs) was created for the first time by combining hexagonal boron nitride (*h*-BN) with doped gadolinium oxide (Gd_2_O_3_) using different laser pulse numbers, i.e., 150, 338, and 772 pulses. We employed various analytical techniques, including mapping analysis, FE-SEM, EDS, HRTEM, SAED, XRD, zeta potential analysis, DLS, FTIR, Raman spectroscopy, and PL measurements, to characterize the synthesized *h*-BN, *c*-Gd_2_O_3_, and *h*-BN@Gd_2_O_3_ NCs (338 pulses). XRD results indicated hexagonal and cubic crystal structures for BN and Gd_2_O_3_, respectively, while EDS confirmed their chemical composition and elemental mapping. Chemical bonds between B–N–Gd, B–N–O, and Gd–O bands at 412, 455, 474, and 520 cm^−1^ were identified by FTIR analysis. The antimicrobial and anticancer activities of these NCs using agar well diffusion and MTT assays. They exhibited potent antibacterial properties against both Gram-positive and Gram-negative pathogens. Furthermore, NCs have reduced the proliferation of cancerous cells, i.e., human colon adenocarcinoma cells (HT-29) and human breast cancer cells (MCF-7), while not affecting the proliferation of the normal breast cell line (MCF-10). The anticancer efficacy of NCs was validated by the AO/EtBr assay, which confirmed apoptotic cell death. Blood compatibility on human erythrocytes was also confirmed by hemolytic and in vitro toxicity assessments. The compiled results of the study proposed these nanoparticles could be used as a promising drug delivery system and potentially in healthcare applications.

## Introduction

Nanotechnology has revolutionized therapeutic approaches by creating materials at the nanoscale. Nanoparticles possess unique properties that differ from larger particles, influencing their interactions within biological systems. Size is important in biological processes such as crossing barriers and immune recognition. Nanotechnology offers advantages over conventional methods by enhancing cellular retention and penetration. Particles larger than 100 nm but with exceptional properties are also considered nanomaterials^1,2^. Currently, there is a pressing need for an advanced treatment approach that can effectively overcome the barrier posed by the cell membrane and ensure targeted drug delivery and sustained retention at the desired site. In comparison to conventional agents, drugs engineered at the nanoscale can offer a multitude of pharmacological advantages. The creation of unique and superior "Nano-drugs" is possible by fusing the extensive understanding of nanoparticles (NPs) with the most recent knowledge of cellular and molecular activities^3^. These NPs possess the remarkable ability to encapsulate, incorporate, or conjugate various drug molecules, enabling precise delivery to specific targets^3–5^. Cancer, among other devastating diseases, remains a major cause of mortality and demands urgent intervention. Breast cancer, in particular, continues to claim a significant number of lives worldwide^6^. The utilization of existing chemotherapy drugs has its drawbacks, including high costs, toxicity, and severe side effects. Therefore, in order to solve this urgent crisis, it is essential to investigate alternative medicines, such as nanomedications and nanoformulations for drug delivery.

Metal oxide nanoparticles (MONPs) and metal nitride nanoparticles (MNNPs) are highly sought-after nanomaterials in the realm of biologically active nanoparticles. This is mainly because of their compact size and high surface-to-volume ratio, making them increasingly utilized in medical and consumer products. Recently, MONPs and MNNPs have attracted great interest as antibacterial and anticancer drugs due to their exceptional performance, enhanced properties, cost-effectiveness throughout their life cycle, and extensive applicability in various industrial sectors and biomedical applications^7^. Despite significant advancements in nanotechnology, the effects of gadolinium oxide (Gd_2_O_3_) and boron nitride (BN) NPs on cancer cell lines are not yet definitively understood. Previous research on NPs has indicated their relative toxicity to cancer cells while sparing healthy cells^4^. Notably, considerable attention is being directed towards Gd_2_O_3_ and BN NPs due to their cost-effectiveness, being approximately 10 times cheaper than silver and gold in the market^8^. Thus, utilizing BN and Gd_2_O_3_ NPs would not only provide an effective method but also prove economically advantageous. Furthermore, BN and Gd_2_O_3_ NPs have demonstrated antibacterial properties against both Gram − ve and Gram + ve microorganisms, displaying high sensitivity even at low concentrations of BN and Gd_2_O_3_ NPs^9–11^.

Gd_2_O_3_ has emerged as a promising candidate with potential antibacterial properties. Recent research has focused on exploring the efficacy of Gd_2_O_3_NPs in inhibiting bacterial growth and eliminating bacterial biofilms^12^. These nanoparticles have shown promising results in combating various types of bacteria, including both Gram + ve and Gram − ve strains. The antibacterial effects of gadolinium oxide nanoparticles can be attributed to their capacity to generate reactive oxygen species (ROS) when exposed to light or heat. This ROS production induces oxidative stress and damages bacterial cells, leading to their demise. Additionally, Gd_2_O_3_NPs have proven effective in disrupting bacterial biofilms, which are notorious for their resilience against antibiotics^9,12^.

BN is a compound comprised of boron and nitrogen atoms structured in a hexagonal lattice formation. It possesses exceptional characteristics that make it a highly promising material for a variety of applications, including those within the medical field^13^. In terms of antibacterial uses, BNNPs have demonstrated the potential to inhibit bacterial growth. These nanomaterials are capable of physically interacting with bacterial cell membranes, resulting in membrane disruption and subsequent bacterial death^11^. Moreover, BNNPs exhibit antibacterial activity by releasing ions, such as borate and nitrate ions, which impede bacterial function and hinder their growth. When considering its applications in the realm of cancer treatment, BN has been examined for its potential as a drug delivery system and photothermal therapy agent. Its remarkable thermal conductivity allows for the efficient conversion of light into heat, which facilitates the targeted destruction of cancer cells during photothermal therapy^14^. Additionally, BNNPs can serve as carriers for anticancer drugs, enhancing their stability and enabling more precise administration to tumor sites. It is important to note that while both Gd_2_O_3_ and BN exhibit promise in antibacterial and anticancer applications, further research is necessary to optimize their efficacy, comprehend their mechanisms of action, and assess potential side effects when used in clinical settings^10,11^.

Scientists are interested in hybridizing elements on the nanoscale due to their unique physicochemical features. This results in a new class of nanoparticles called "core–shell nanoparticles" that combine different types of nanoparticles. The surface-to-volume ratio increases, altering the characteristics, and the shell layer can enhance the core material's attributes^15^. The study found that Gd_2_O_3_ is the best shell material for h-BN due to its ability to undergo cell transition. This structure has potential applications in medicine and pharmaceuticals. Currently, a large number of chemical, physical, biological, and hybrid methods are available to synthesize different types of nanoparticles^16^. The nanoparticles formed using each method show specific properties^17^.

Pulse laser deposition (PLD)^18,19^, sol–gel preparation^20,21^, a high-speed stirring method^22,23^ and hydrothermal procedures^13,24, 25^ are a few of the methods that may be used to create NPs. However, a hybrid technique combining pulse laser ablation in liquid (PLAL) with laser fragmentation in liquid has gained favor since it is economical and ecologically beneficial. This hybrid approach has the benefit of requiring less expensive equipment and only a few minutes of setup time.

The process of creating nanomaterials through PLAL (Pulsed Laser Ablation in Liquid) involves absorbing a laser pulse from a material, which leads to the formation and cooling of a plasma plume. This leads to shock waves, creating bubbles in a liquid. Laser fragmentation, on the other hand, exposes micro- or Nano-powders suspended in a liquid to laser radiation. This melts particles in the laser beams' focus, interacting with the liquid's vapors and causing fragmentation. This method is used to reduce nanoparticle size and modify their morphology^26–28^.

Hybrid techniques involve combining two or more techniques to produce a new composition in suspension. An innovative method known as laser exfoliation and fragmentation in liquid (LEFL) has been developed, which utilizes a laser beam and probe sonication to separate BN layers into atomically thin sheets^29^. This process also creates binding sites on the surface of these sheets, enabling interaction with other two-dimension (2D) nanomaterials like graphene and NPs such as Gd_2_O_3_. By employing laser ablation in liquid, researchers can achieve precise control over the synthesis process, allowing for the production of hybrid nanocomposites (HNCs) from BN and Gd_2_O_3_ with tailored properties for specific applications, such as drug delivery systems or biomedical imaging^30^.

Currently, there is a research gap regarding the synthesis of the BN, Gd_2_O_3_, and BN@Gd_2_O_3_ NCs. To address this gap, we propose a novel two-step PLAL method that combines probe sonication and fragmentation. Our study aimed to investigate the influence of laser pulse quantity on the structural, morphological, and optical properties of the core–shell nanoparticles BN@Gd_2_O_3_. Therefore, the best conditions for synthesizing BN, Gd_2_O_3_, and BN@Gd_2_O_3_ were identified and subsequently analyzed. The primary objective of our research is to synthesize BN@Gd_2_O_3_ core–shell NPs for their potential use as an antibacterial agent. Additionally, this study is the first to investigate the effects of BN@Gd_2_O_3_ nanoparticles on Gram-ve bacteria (*Escherichia coli* and *Proteus mirabilis*) and Gram + ve bacteria (*Streptococcus mutans* and *Staphylococcus aureus*). A cytotoxic assay using MCF-10, MCF-7, and HT-29 to evaluate the impact of the synthesized *h*-BNNs, *c*-Gd_2_O_3_Ns, and *h*-BN@Gd_2_O_3_ NCs was also conducted.

## Materials and methods

### Materials

The analytical-grade chemical materials of boron nitride and gadolinium oxide were purchased from Xian Lyphar Biotech Co., Ltd., China, and Sky Spring Nanomaterial, Inc., USA, respectively. From Sigma Chemical Co., St. Louis, USA, fetal bovine serum and 3-(4,5-dimethylthiazal-z-yl)-2,5-diphenyltetrazolium (MTT) were obtained. Dulbecco's Modified Eagle Medium (DMEM) was purchased from Euro Clone, Milan, Italy. The antibiotics penicillin and streptomycin (BioSource International, Nivelles, Belgium). Muller-Hinton agar medium, obtained from Mast, Liverpool, England.

### Synthesis of ***h***-BN and Gd_2_O_3_ NPs

The section on materials and methods describes the synthesis of *h*-BN and Gd_2_O_3_ nanoparticles. In our previous work^29^, we demonstrated laser exfoliation and fragmentation of BN in a liquid. To synthesize the nanoparticles, an Nd: YAG laser with 2 ns pulse duration at 3 Hz was used to focus on the Gd_2_O_3_ target and BN powder. The resulting ablated samples were collected by centrifuge to remove large particles before further use in vitro.

### Synthesis of ***h***-BN@Gd_2_O_3_ hybrid NCs

**Figure 1:**
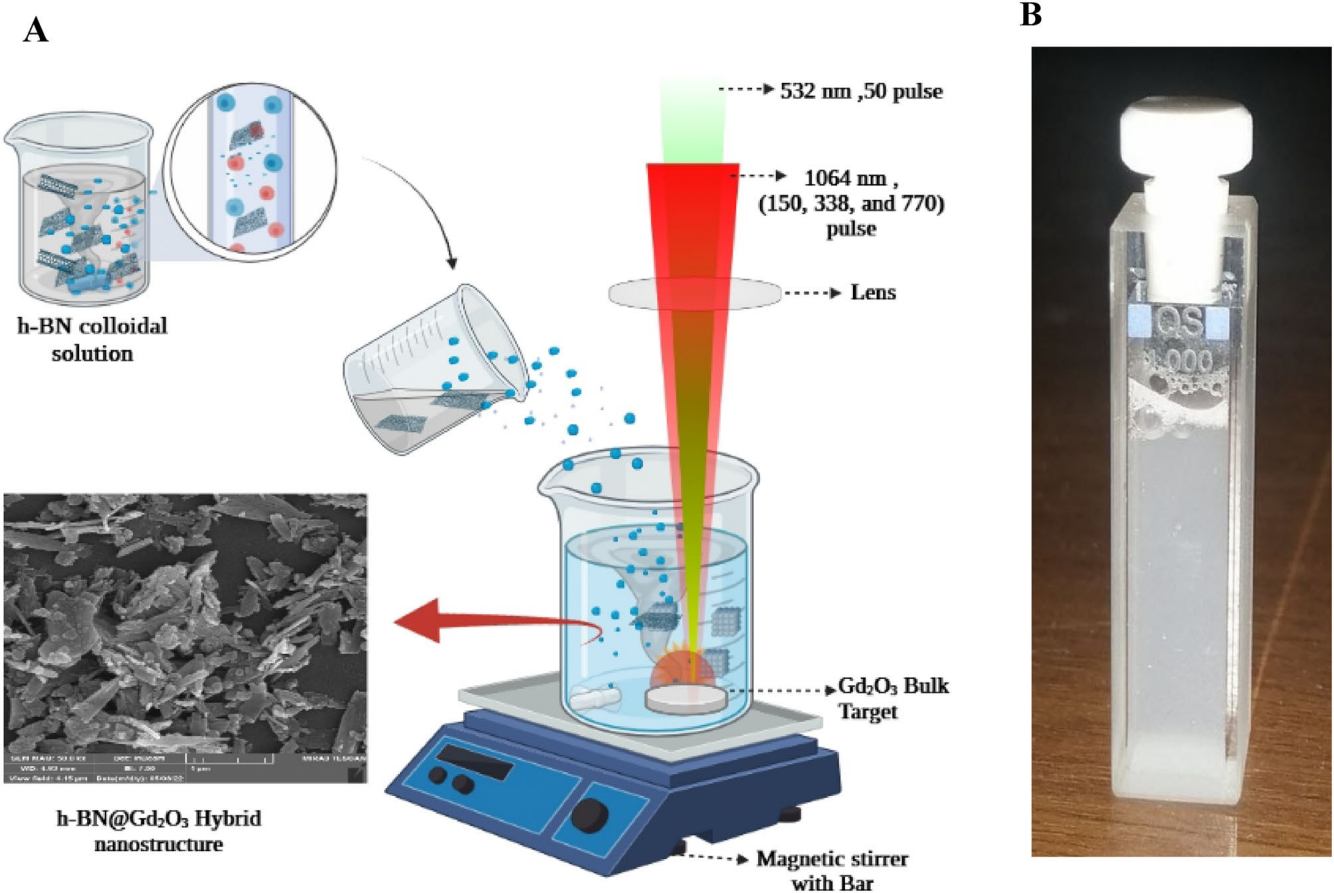
(**A**) Illustration of the hybrid approach to preparing *h*-BN@*c*-Gd_2_O_3_ colloids. (**B**) Photographs for the prepared colloids of *h*-BN@c-Gd_2_O_3_ NCs.

**Figure 2:**
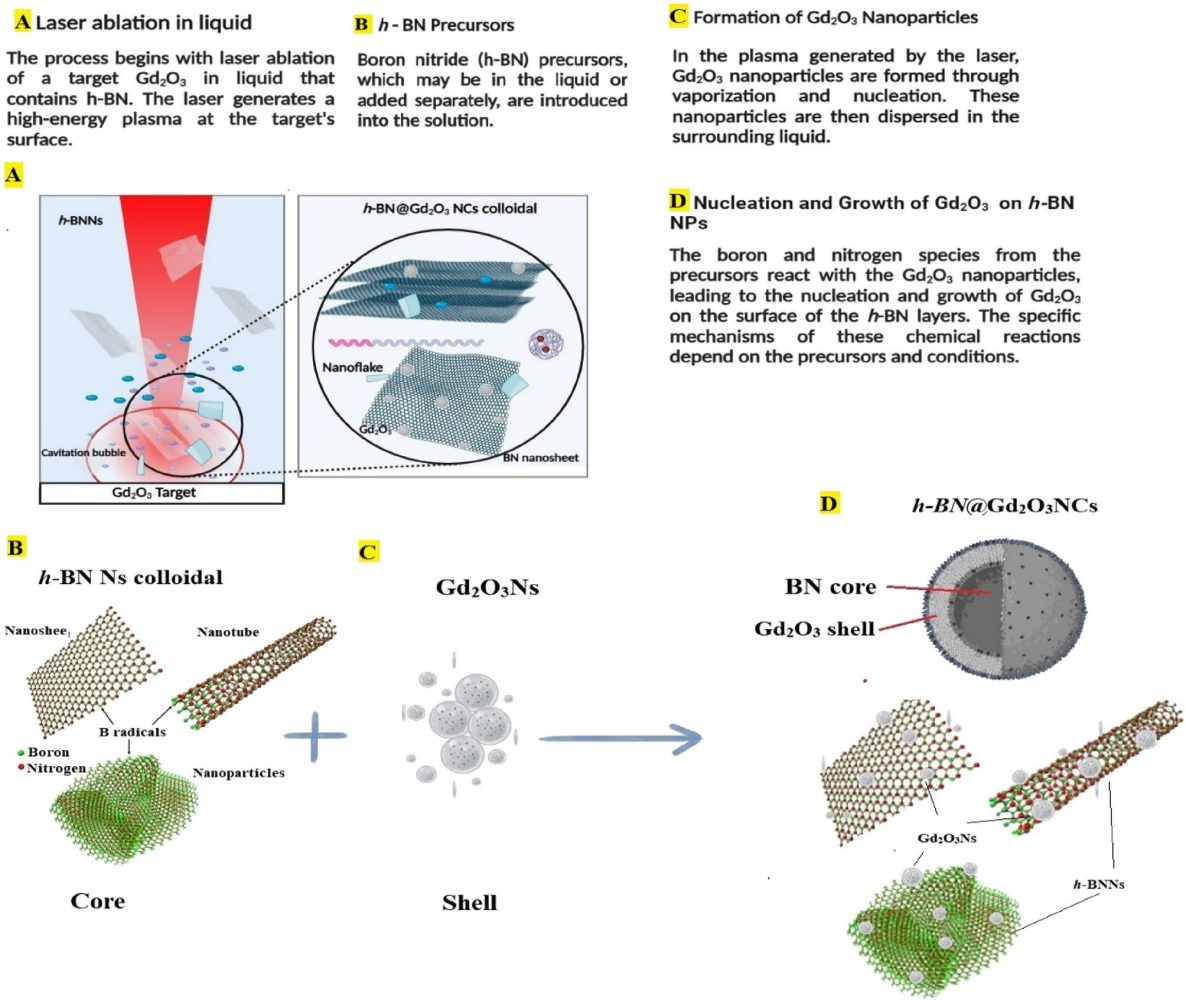
Illustration of the mechanism for the formation of *h*-BN@*c*-Gd_2_O_3_ NCs colloids.

To create *h*-BN@Gd_2_O_3_ HNCs, we followed a two-step process. First, we prepared an *h*-BN suspension using similar steps as described in "Synthesis of *h*-BN and Gd_2_O_3_ NPs". In the second step, we ablated the Gd_2_O_3_ pellet using the same laser with a fluence of 1.27 Jcm^-2^ in colloid *h-*BN Ns solution at different numbers of laser pulses (150, 338, and 772). A schematic diagram of the procedure for the formation of *h*-BN@Gd_2_O_3_ NCs via the hybrid technique is shown in Fig. 1. Figure 2 shows a schematic diagram of the mechanisms of the formation of *h*-BN@Gd_2_O_3_ NCs via laser ablation in liquid technique. Selecting the appropriate parameter, such as the number of laser pulses, is crucial in antibacterial and cancer treatment experiments. This choice enables us to achieve the necessary high concentration for optimal results in eliminating bacteria or cancer cells. In addition, it helps us determine the ideal parameter for synthesis, which is 10 mJ with 338 pulses, as it yields the most favorable outcome.

### Characterization of ***h***-BNNs, ***c***-Gd_2_O_3_Ns, and ***h***-BN@Gd_2_O_3_ NCs

Several methods were used to examine the materials' morphology, structure, chemical, and optical properties. Field Emission Scanning Electron Microscopy (FE-SEM) with Energy Dispersive Spectroscopy (EDS) and mapping analysis were conducted using the Inspect F50 system (a small amount, one drop, of the prepared sample was deposited on a glass substrate). Transmission electron microscopy with high resolution (HR-TEM) from JEOL in Tokyo, Japan, was employed with Selected Area Electron Diffraction (SAED). A small amount (one drop) of the prepared sample was deposited on a gold mish, and then the samples underwent a coating process with a thin layer of gold to enhance imaging quality and reduce charging effects. X-ray crystallography (XRD) data were collected using a Shimadzu X-ray diffractometer at 40 kV and 25 mA with Cu-Kα radiation at 1.5405 Å. Nano plus DLS Nano Particle Sizer was used to assess the particle size and zeta potential (the colloidal sample was placed in a glass cuvette). Instruments of various types were used to evaluate chemical and optical qualities. A Perkin-Elmer spectrometer was used to collect FT-IR spectra in the 400–4000 cm^−1^ range. A THERMO SCIENTIFIC DXR FT-Raman spectrometer was used to record the Raman spectra. The laser source had a 2-mW output at 532 nm. Using a Shimadzu UV-2600 spectrometer, the UV absorbance spectra of the colloidal suspensions were determined between 200 and 500 nm. Shimadzu Spectra fluorophotometer model RF-5301pc was used to measure Photoluminescence Spectroscopy) PL (, and the colloidal sample was placed in a quartz cuvette.

### Antibacterial activity of ***h***-BNNs, ***c***-Gd_2_O_3_, and ***h***-BN@Gd_2_O_3_ NCs

Using an agar well diffusion experiment, the antimicrobial activity of the produced materials (*h*-BNNs, *c*-Gd_2_O_3_Ns, and *h*-BN@Gd_2_O_3_ NCs) was examined against both Gram-ve (*Escherichia coli* and *Proteus mirabilis*) and Gram + ve (*Streptococcus mutans* and *Staphylococcus aureus*) bacterial isolates, which were identified using the VITEK system (VITEK, Biomérieux, Marcy-l’Etoile, France), kindly provided by the Medical Microbiology Laboratory, Biotechnology Division, Department of Applied Science, University of Technology, Baghdad, Iraq. The McFarland standard was used to obtain suspensions at 1.5 × 10^8^ colony-forming units (CFU) mL^-1^. Muller-Hinton (MH) agar was aseptically put onto sterile Petri plates in an amount of 20 mL. Using a sterile wire loop, the bacteria were extracted from their stock cultures. Using a sterile point, 6 mm-diameter wells were drilled into the agar plates after the organisms had been cultured. In the dreary wells. Before measuring and recording the average zones of inhibition diameter, the cultivated plates containing the test organisms and samples (*h*-BNNs, *c*-Gd_2_O_3_Ns, and *h*-BN@Gd_2_O_3_ NCs at a concentration of 50 µg mL^−1^ of each) were incubated overnight at 37 °C for 24 h in an upright position, and inhibition zones were measured in millimeters by measuring the diameter of circular inhibition zones around the well using a physical ruler. Deionized distilled water (DDW) was used as a control. The assay for antibacterial activity was performed in triplicate^31^.

### Culturing of cell lines

The human colon adenocarcinoma cells (HT-29), Michigan Cancer Foundation-7 (MCF-7; human breast cancer cells), and normal breast MCF-10 were provided by the American Type Culture Collection (ATCC, Manassas, USA). The cells were grown in DMEM supplemented with 10% FBS, 2 mM l-glutamine, and 20 mM HEPES and employed using tissue culture-treated flasks (T 25 cm^2^; Falcon, USA) under the conditions of 5% CO_2_ and 37 °C.

### MTT assay

To assess the cell viability and cytotoxicity of *h*-BNNs, *c*-Gd_2_O_3_Ns, and *h*-BN@Gd_2_O_3_ NCs, cells were seeded (MCF-7, MCF-10, and HT-29: 4–6 × 10^4^ cells per well in 100 μl culture media) in 96-well plates (SPL Life Sciences, Korea) and cultivated overnight. Afterward, cells with 70–80% of confluence were treated with diverse concentrations of *h*-BNNs, *c*-Gd_2_O_3_Ns, and *h*-BN@Gd_2_O_3_ NCs (1, 5, 10, 15, 25, 50, and 75 µg/ml) and incubated for 24 h. Prior to conducting the experiment, the plates were incubated at 37 °C for 24 h with 20 μL of MTT (5 mg/ml in PBS), specifically thiazolyl blue tetrazolium bromide solution. Subsequently, the plates were kept in the dark for 4 h. The change from yellow MTT dye to purple formazan indicates the metabolic activity of the cells. Afterward, the MTT solution was removed, and each well was treated with 100 μL of dimethyl sulfoxide (DMSO) to dissolve the formazan. Using a Sat Fax 2100 microplate reader from Stat Fax, USA. Absorbance at a wavelength of 545 nm was measured to quantify the extent of dye conversion. The proportion of dye conversion by untreated cells was utilized to determine the viability and/or metabolic activity of the cells^32^. The formula (1) below was applied to determine the inhibition percentage:

1 $$Inhibition rate\left(\%\right)=\left(Abc-\frac{Abs}{Abc}\right){\times}100$$

where *Abc* and *Abs* were the optical density of the control and tested samples, respectively.

### Dual staining assay

To assess the apoptosis effects of *h*-BNNs, *c*-Gd_2_O_3_Ns, and *h*-BN@Gd_2_O_3_ NCs in HT-29 and MCF-7 cells, dual-labeling using AO/EtBr dye was carried out according to the method of Al-Jubori and his co-workers^33^. Briefly, cells were seeded (density 1 × 10^5^ cells per mL) using DMEM in 96-well microtiter plates and incubated overnight. IC_50_ concentrations of h-BNNs, *c*-Gd_2_O_3_Ns, and *h*-BN@Gd_2_O_3_ NCs were then applied for 24 h, followed by washing with PBS, applying dual fluorescent dyes (100 μL) to the cells, and finally visualized under a fluorescent microscope.

### Hemocompatibility test

Fresh samples of blood were taken from five healthy donors and distributed into heparin-coated tubes based on the method of the NIH (National Institute of Health) and FDA (Food and Drug Administration) and as per the declaration and regulation of Helsinki of 1975 as a statement of ethical principles. Permission was obtained from the hospitals of the medical city in Baghdad, Iraq, and approved by the institutional ethical committee of the Department of Applied Sciences, University of Technology, Baghdad, Iraq (Ref. No. 216AS18/1/2021). Study participants were informed about the value of the study before we collected any samples. Informed consent was obtained from the study participants.

A hemolysis test was performed for the *h*-BNNs, *c*-Gd_2_O_3_Ns, and *h*-BN@Gd_2_O_3_NCs samples based on a previously described method^33^. Briefly, whole blood (100 μL) was mixed with PBS (700 μL) and 100 μL of *h*-BNNs, *c*-Gd_2_O_3_Ns, or *h*-BN@Gd_2_O_3_NCs at seven concentrations (1, 5, 10, 15,25,50, and 100 μg mL^−1^) were added; phosphate buffer saline served as a negative control (0% hemolysis), whereas deionized distilled water served as a positive control (100% hemolysis). After shaking for 1 h at 37 °C, the reaction mixture is centrifuged at 700 rpm for 5 min, and the absorbance of the supernatant at 541 nm is measured. The hemolysis value percentages were therefore expressed in the following equation (2).

2 $$Percentage \;hemolysis \left(\%\right)=\left(\frac{OD \;sample-OD\left(-ve\right)control}{OD\left(+ve\right)control-OD\left(-ve\right)control}\right) \times 100$$

### Statistical analysis

We utilized Graph Pad Prism version 8, Image J, and Origin 2021 software to analyze the results obtained from the conducted experiments. A one-way ANOVA was used to analyze the data, and then significant differences were set at * p ≤ 0.05, ** p ≤ 0.01, or *** p ≤ 0.01. Data were presented as mean ± standard deviation.

## Results and discussion

**Figure 3:**
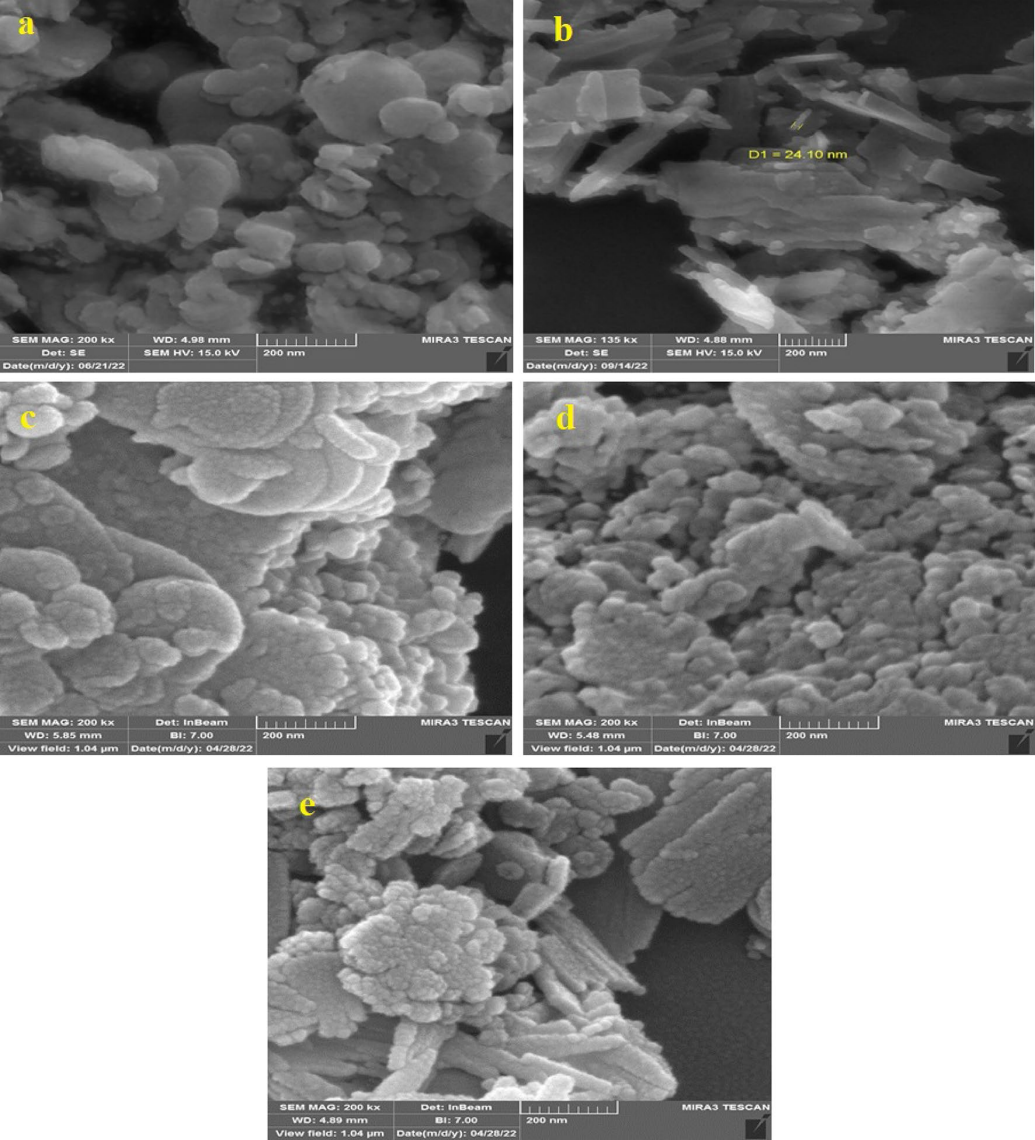
The FESEM of (**a**) *h*-BNNs, (**b**) *c*-Gd_2_O_3_Ns, (**c**) *h*-BN@Gd_2_O_3_NCs at (150 pulse), (**d**) *h*-BN@Gd_2_O_3_ NCs at (338 pulse), and (**e**) *h*-BN@Gd_2_O_3_NCs at (772 pulse).

**Figure 4:**
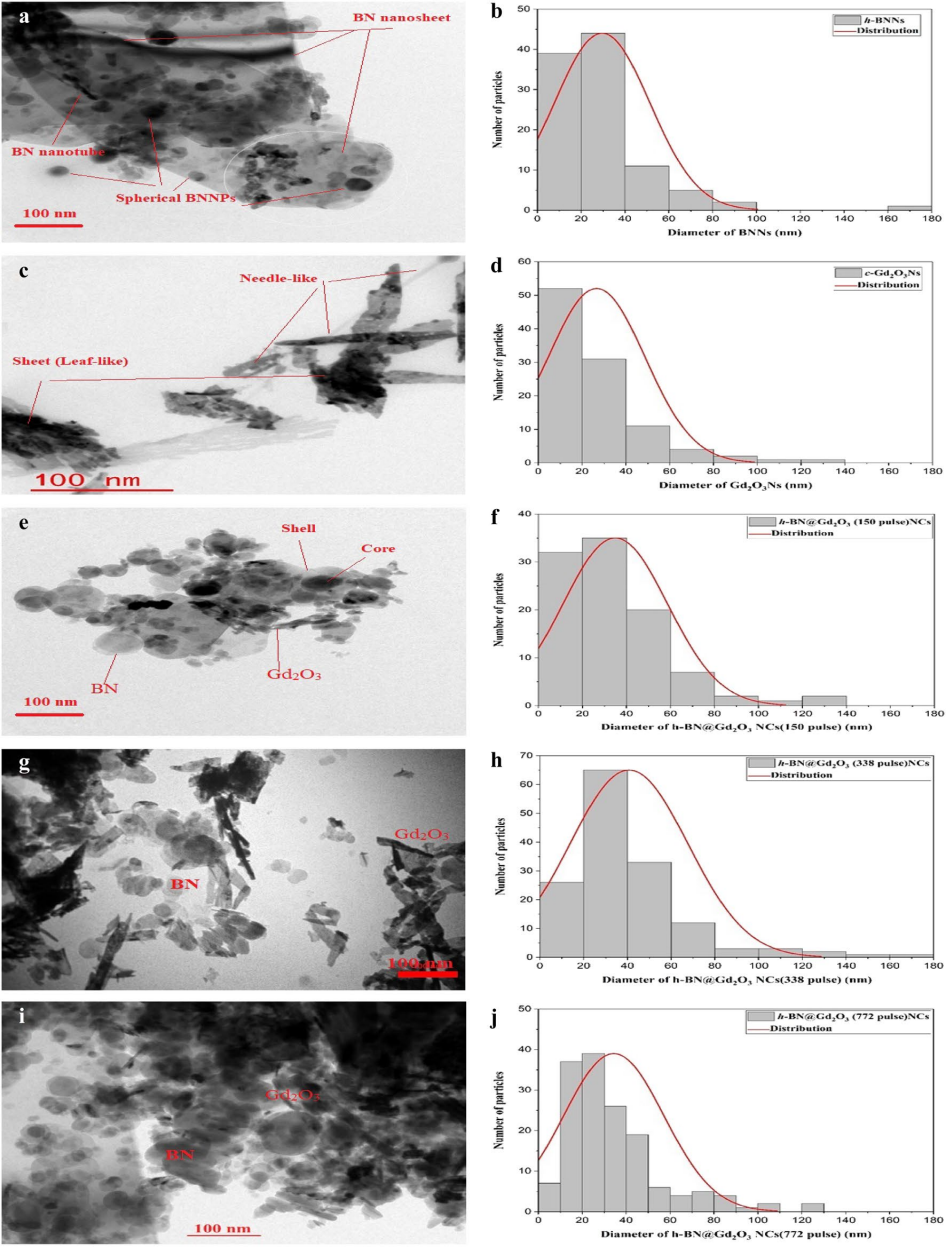
The HRTEM and particle size of (**a,b**) *h*-BNNs, (**c,d**) *c*-Gd_2_O_3_Ns, (**e,f**) *h*-BN@Gd_2_O_3_NCs at (150 pulse), (**g,h**) *h*-BN@Gd_2_O_3_ NCs at (338 pulse), and (**i,j**) *h*-BN@Gd_2_O_3_NCs at (772 pulse).

Figures 3 and 4 illustrate the morphology, structure, and particle size of the BNNs, Gd_2_O_3_Ns, and *h*-BN@Gd_2_O_3_ obtained with varying numbers of laser pulses. The FESEM analysis revealed that the structures change as the number of laser pulses increases in DDW. In Fig. 3a, the h-BNNs exhibit small particles with irregular spherical shapes and nanosheets, these results validate those of Guerra et al., Thangasamy et al., and Kumar et al.^34–36^. On the other hand, Gd_2_O_3_Ns (Fig. 3b) display nanofiber-like, needle-like, and sheets (leaf-like) morphologies with significant heterogeneity, clearly demonstrating the flaky structure of the prepared Gd_2_O_3_. The Gd_2_O_3_ nanocrystalline flakes are flat and have irregular shapes, as observed in the micrographs, these results validate those of Almeida et al., and Jeon et al.^37,38^. However, in the case of the nanocomposites during the synthesis of *h*-BN@Gd_2_O_3_, the *h*-BN and *c*-Gd_2_O_3_ particles tend to coalesce, as depicted in Fig. 3c–e. The results indicated that the h-BNNs consisted of spherical-shaped particles, while Gd_2_O_3_ exhibited numerous flake-like and sheets (leaf-like) morphologies with a layered structure, making it easy to identify the Nano type. Most of the nanostructures are interconnected, forming networks that do not exhibit well-ordered patterns, and their sizes and thicknesses vary.

The HRTEM was used to capture images of *h*-BNNs, *c*-Gd_2_O_3_Ns, and *h*-BN@Gd_2_O_3_NCs, which were synthesized using different numbers of laser pulses, resulting in a variety of morphologies. The TEM analysis findings were consistent with those obtained from the FESEM images. Specifically, Fig. 4a shows well-dispersed and uniform h-BNNs with both spherical and nanosheet shapes^36^. These NPs' average particle size was found to be 30 nm. On the other hand, Fig. 4b reveals the presence of diverse Gd_2_O_3_ nanostructures, including needle-like and sheet-like structures. These NPs' average particle size indicated that these Gd_2_O_3_Ns had varying lengths ranging from 40 to 140 nm and diameters ranging from 5 to 30 nm. It is interesting that the number of laser pulses had a considerable impact on the size and morphology of the synthesized *h*-BN@Gd_2_O_3_ NCs, as observed in Fig. 4c–e. As the number of laser pulses increased, the average crystallite size of the Ns decreased. The core–shell structure of the prepared *h*-BN and *c*-Gd_2_O_3_ NPs was confirmed by the TEM images. In this structure, the dark points represent the h-BNNs (core) surrounded by the light points, which represent the Gd_2_O_3_Ns (shell).

**Figure 5:**
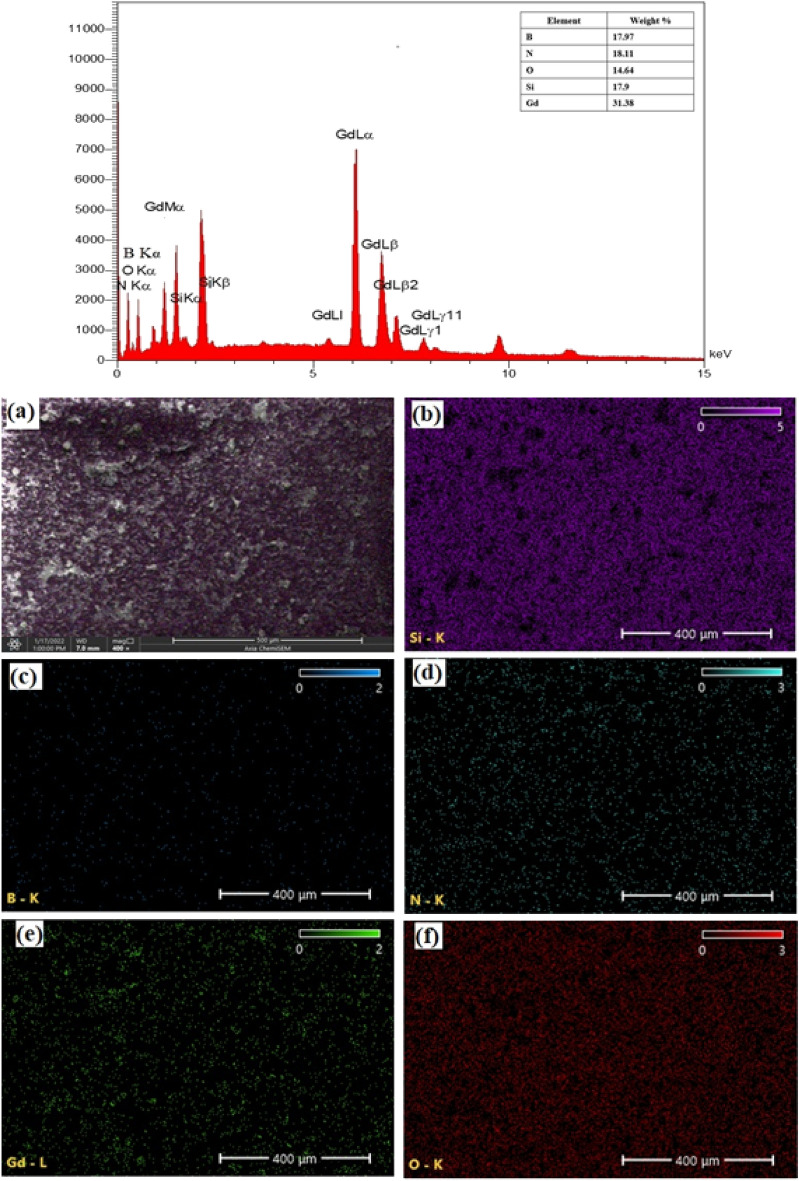
EDS spectrum and analyzed weight % of B, N, O, Si, and Gd (inset) for the *h*-BN@Gd_2_O_3_ NCs at (338 pulses). (**a**) FE-SEM image of *h*-BN@Gd_2_O_3_ NCs at (338 pulses), and corresponding EDS elemental mapping for (**b**) Si, (**c**) B, (**d**) N, (**e**) Gd, and (**f**) O.

The *h*-BN@Gd_2_O_3_ NCs, synthesized with 338 pulses, underwent EDS analysis to determine their chemical composition. Figure 5 (left lane) presents the EDS spectra of the *h*-BN@Gd_2_O_3_ NCs deposited on a glass substrate along with the corresponding weight percentages of elements. The analysis revealed the presence of boron (B), nitrogen (N), gadolinium (Gd), oxygen (O), and silicon (Si) elements in the *h*-BN@Gd_2_O_3_ NCs. The O elements can be attributed to interactions with the atmosphere, Gd_2_O_3_ target, and DDW. As a result, the prepared NCs are obviously devoid of additional contaminants. Due to the poor electron scattering by light components, this method is not suited for directly measuring the presence of hydrogen (H) in the Nanocomposite. Nevertheless, micro-Raman, FTIR, and XRD analyses can be used to infer the existence of the H element. The findings of the EDS mapping and spectrum show that the *h*-BN@Gd_2_O_3_ NCs (core–shell) successfully formed and that the distribution of the elements in the sample was uniform. In the right lane of Fig. 5a, the SEM image of the sample is shown, while Fig. 5b–f correspond to the formation of silicon (from the glass substrate), boron, nitrogen, gadolinium, and oxygen nanoparticles in the sample, respectively.

**Figure 6:**
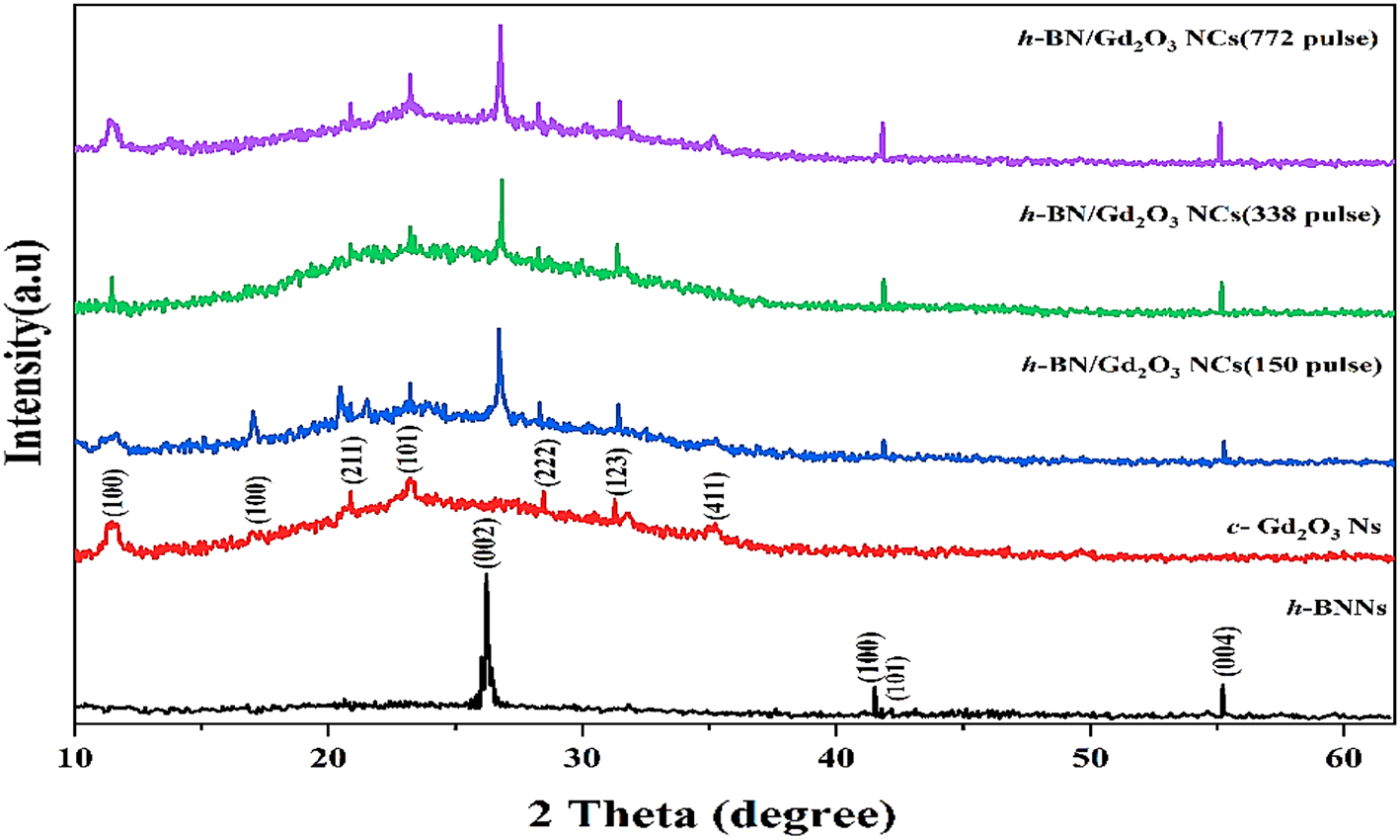
XRD pattern of the prepared sample, *h*-BN Ns, *c*-Gd_2_O_3_ Ns, and *h*-BN@Gd_2_O_3_ NCs with different laser pulses.

Figure 6 shows the XRD pattern for all samples that were synthesized using the hybrid approach. For determining the physical structure of nanoparticles, XRD is a valuable technique. We can see that the diffraction peaks of the h-BNNs were found at 2 = 26.225°, 41.525°, 42.125°, and 55.225°, and these peaks corresponded to the Miller indices (002), (100), (101), and (004), respectively. The creation of *h*-BN is indicated by the strongest diffraction pattern between 25 and 30°, which is compatible with JCPDS 34-0421 and JCPDF 45-0893^34,36^. The diffraction peaks of Gd_2_O_3_Ns were observed at 2θ = 11.525°, 17.025°, 20.875°, 23.225°, 28.475°, 31.275°, and 35.225°, corresponding to (100), (211), (101), (222), (123), and (411) planes, respectively, according to JCPDS 03-065-3181, JCPDS 88-2165, and JCPDS 12-0797, which indicates cubic phase crystallites of Gd_2_O_3_^37–39^. Using Debye–Scherrer Eq. (3), the average crystal size was calculated.

3 $$The \;typical \;crystallite \;dimension \left(D\right)=\frac{0.9\lambda }{\beta cos\theta }$$

where is Bragg's diffraction angle, is the angular peak width at half maximum (in rad), and is the X-ray wavelength (= 1.54060). The average crystallite sizes for *h*-BNNs, *c*-Gd_2_O_3_Ns, *h*-BN@Gd_2_O_3_ (150 pulses), *h*-BN@Gd_2_O_3_ (338 pulses), and *h*-BN@Gd_2_O_3_ (772 pulses) were determined to be 7.04 nm, 0.68 nm, 2.59 nm, 3.75 nm, and 4.88 nm, respectively. Table 1 provides the calculated crystallite size for each sample. These findings corroborate the FESEM results, indicating the successful formation of nanometer-sized particles. These results are in full agreement with previously reported data conducted by other researchers^29,34, 37, 39^. When compared to the XRD patterns of h-BNNs and *c*-Gd_2_O_3_, the diffraction patterns of *h*-BN@Gd_2_O_3_ produced at different laser pulse levels correspond to both the *h*-BNNs peak and the *c*-Gd_2_O_3_Ns peak. This confirmation supports the creation of a hybridized material (core/shell) without any chemical interaction between them. A slight increase in the intensity of the diffraction peaks has been observed, but there has not been a significant broadening of the peak. This is because more laser pulses lead to a higher quantity of NCs in the colloidal solution^40–42^. This observation implies an enhancement in crystallinity due to doping. The data indicate that both BN and Gd_2_O_3_ influenced the crystallite sizes of the NCs.

**Table 1:** XRD peaks details of *h*-BNNs, *c*-Gd_2_O_3_Ns, and *h*-BN@Gd_2_O_3_NCs.

Element	2θ (deg)	β = FWHM (deg)	D (nm) = 0.9λ/βcosθ	Average (nm)	*h*	*k*	*l*
BNNs	26.225	0.32779	24.91676	7.04	0	0	2
	41.525	6.29929	1.350465		1	0	0
	42.125	30.37237	0.28065		1	0	1
	55.225	5.53852	1.620824		0	0	4
Gd_2_O_3_Ns	11.475	10.73635	0.744625	0.68	1	0	0
	17.025	21.44512	0.375056		1	0	0
	20.875	10.14241	0.797478		2	1	1
	23.225	8.71111	0.932227		1	0	1
	28.475	10.51972	0.780114		2	2	2
	31.275	11.93328	0.692203		1	2	3
	35.225	20.16724	0.413825		4	1	1
BN@Gd_2_O_3_ (150 pulse)	26.721	3.27965	2.492883	2.59	0	0	2
	23.202	3.00443	2.702807		1	0	1
BN@Gd_2_O_3_ (338 pulse)	26.831	2.35328	3.475001	3.75	0	0	2
	23.202	2.02226	4.015504		1	0	1
BN@Gd_2_O_3_ (772 pulse)	26.778	1.83545	4.454902	4.88	0	0	2
	23.202	1.5314	5.302595		1	0	1

**Figure 7:**
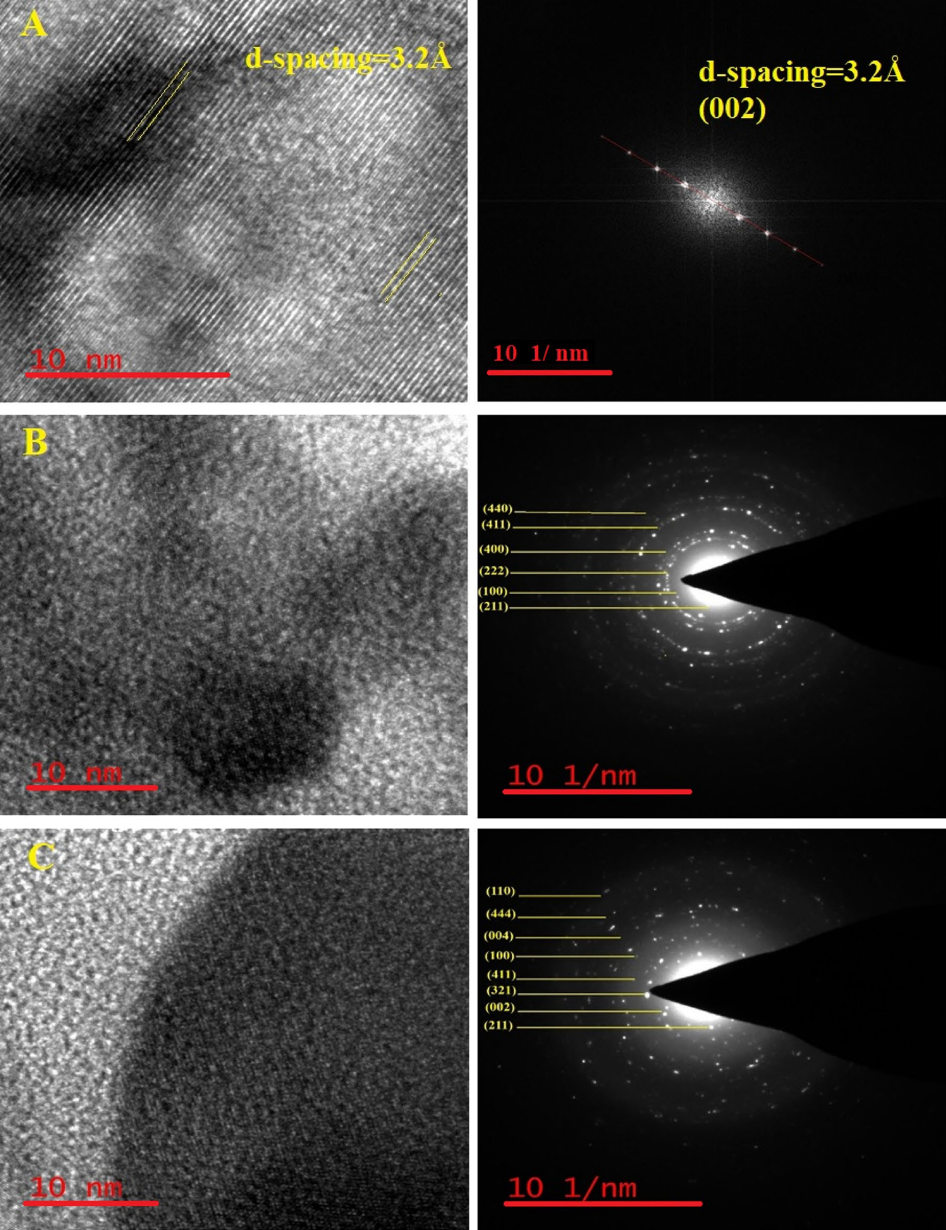
HR-TEM micrographs “interplanar lattice spacing” and SAED patterns for sample synthesized (**A**) *h*-BNNs, (**B**) *c*-Gd_2_O_3_Ns, and (**C**) *h*-BN@Gd_2_O_3_ NC (338 pulses). The TEM results are in good agreement with the XRD results.

The insights garnered from HRTEM and SAED analyses provide a pivotal understanding of the structural attributes of various materials, including *h*-BNs, *c*-Gd_2_O_3_ Ns, and h-BN@Gd_2_O_3_NCs, as illustrated in Fig. 7. In Fig. 7A, the presence of a 3.2 Å d-spacing and the alignment with hkl (002) planes affirm crystallinity, validating the successful synthesis of the hexagonal phase with anticipated interplanar distances. SAED patterns further verify crystallographic orientation and atomic arrangement. The congruence among d-spacing, hkl planes, and SAED reinforces the high-quality synthesis of h-BN through laser exfoliation and fragmentation in deionized water (DDW). Both c-Gd_2_O_3_Ns and *h*-BN@Gd_2_O_3_ NC samples exhibit robust crystallinity, evident from well-defined lattice fringes across a significant proportion of particles (Fig. 7B, C). HRTEM images vividly capture regular lattice fringes, reflecting consistent atom spacing, while SAED patterns present concentric rings, indicating a polycrystalline nature of the sample. The d-spacing values deduced from HRTEM and SAED analyses were cross-referenced with those derived from XRD data, resulting in alignment and confirming the cubic crystal structure for c-Gd_2_O_3_Ns (hkl (100), (211), (222), (123), and (411)) and for *h*-BN@Gd_2_O_3_ NCs (hkl (100), (211), (002), (411), (123), (100), (004), (444), (110), and (411)). These findings collectively endorse the credibility of HRTEM and SAED methodologies in characterizing nanoscale crystal structures. Notably, these results harmonize seamlessly with the XRD findings discussed earlier, solidifying their reliability. These revelations hold promise for potential applications spanning electronics, optoelectronics, and nanocomposites.

**Figure 8:**
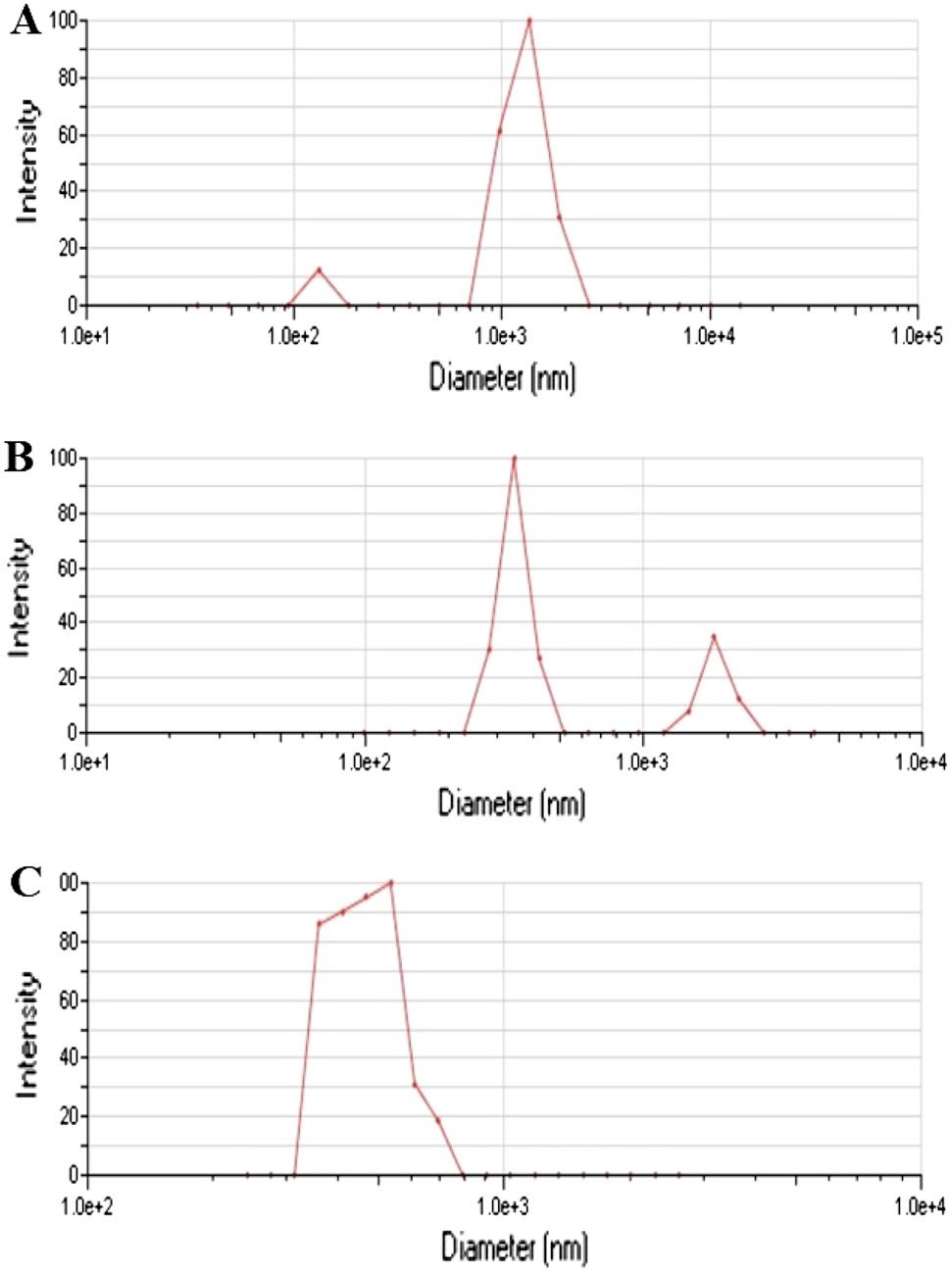
DLS measurement to determine the size distribution used in stability tests of (**A**) *h*-BN, (**B**) *c*-Gd_2_O_3_, and (**C**) *h*-BN@Gd_2_O_3_ NCs (338 pulses).

**Figure 9:**
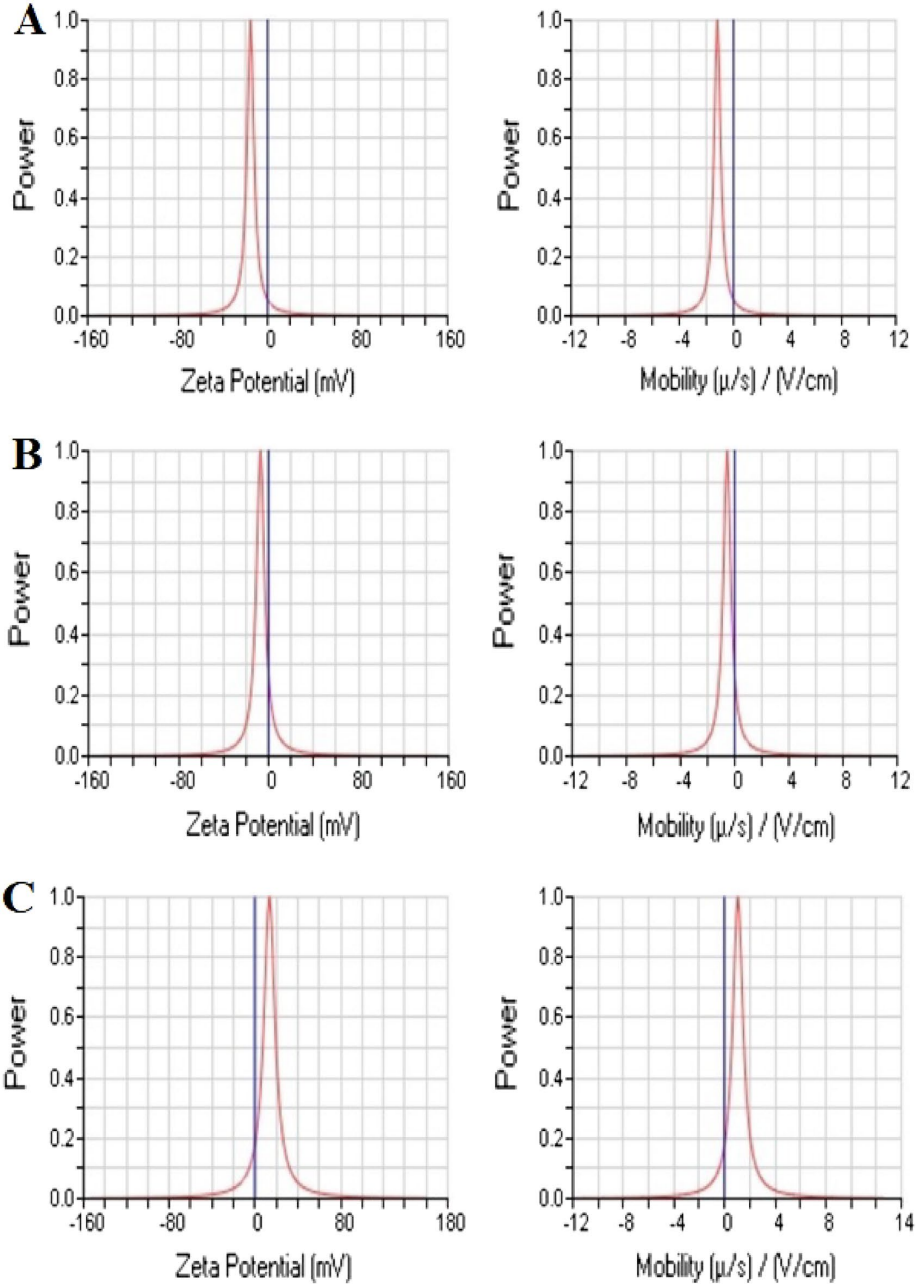
Zeta potential distribution curves were used in stability tests of (**A**) *h*-BN, (**B**) *c*-Gd_2_O_3_, and (**C**) *h*-BN@Gd_2_O_3_ NCs (338 pulses).

Using DLS, it was possible to ascertain the hydrodynamic diameter of *h*-BNNs, *c*-Gd_2_O_3_Ns, and *h*-BN@Gd_2_O_3_ NCs (338 pulses) in DDW medium, which provides information about their particle size (Fig. 8). The hydrodynamic size of *h*-BNNs was measured at 981 nm with a polydispersity of 0.332 (Fig. 8A). For *c*-Gd_2_O_3_ Ns, the hydrodynamic size was 432 nm with a polydispersity of 0.240 (Fig. 8B). As for *h*-BN@Gd_2_O_3_ NCs (338 pulses), the hydrodynamic size was recorded as 573.64 nm with a polydispersity of 0.241 (Fig. 8C). It's crucial to remember that the DLS particle size was discovered to be greater than the results from XRD and TEM, as it represents the hydrodynamic size. Furthermore, the results indicate that h-BNNs exhibited higher dispersibility in DDW compared to Gd_2_O_3_ Ns, as evidenced by their respective hydrodynamic sizes. The value of the zeta potential (ζ) provides important details regarding the potential stability of the colloidal system. Charged particles are repelled by an increase in zeta potential, which prevents them from aggregating in the suspension. A ζ value of at least 30 mV is often displayed by stable systems^41^. When a ζ nanoparticle's value is between −10 and + 10 mV, it is said to be virtually neutral, however when it ζ exceeds + 30 mV or drops below −30 mV, it is classified as strongly cationic or strongly anionic. In our study, we measured ζ of three samples: *h*-BNNs (− 15.20 mV, mobility (μ/s)/(V/cm): −1.19, as shown in Fig. 9A), c-Gd_2_O_3_Ns (−7.17 mV, mobility (μ/s)/(V/cm): −0.56, as shown in Fig. 9B). This negative charge may play an important role. h-BN@Gd_2_O_3_ NCs (338 pulses) (13.53 mV, Mobility (μ/s)/(V/cm): 1.06, indicating incipient instability, as shown in Fig. 9C. The zeta values and the results show that the h-BNNs sample has a high modulus value of ζ = −15.20 mV. This is explained by the fact that the laser exfoliation and fragmentation process leaves hydroxyl groups (OH) on the surface of the BNNs, which improves the suspension's stability^43^. Understanding the zeta potential is important as it helps to comprehend the in vivo destination of materials as it relates to cellular processes like aggregation, adhesion, and activation. This evaluation of stability in the synthesized materials is essential when they are dispersed in DDW. However, it is important to note that other factors such as particle size, surface charge density, and the presence of stabilizing agents also play significant roles in determining the overall stability of nanoparticles^44^.

**Figure 10:**
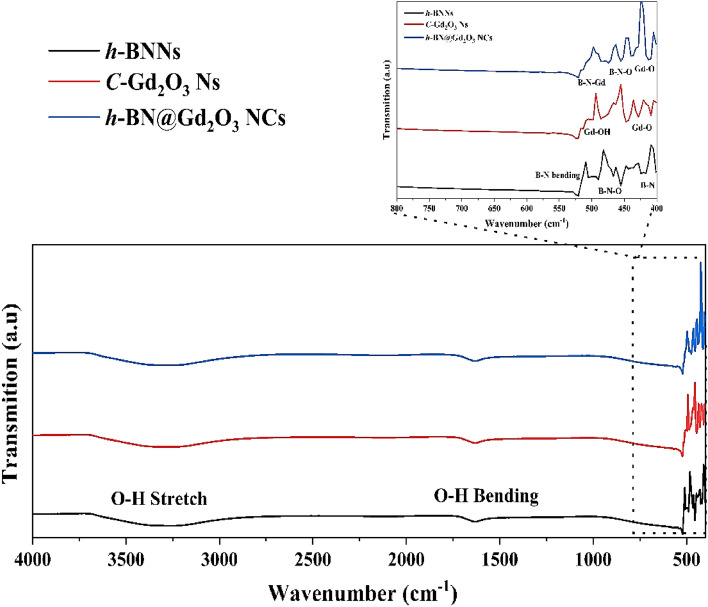
FTIR spectra of the prepared samples, *h*-BN Ns, *c*-Gd_2_O_3_ Ns, and *h*-BN@Gd_2_O_3_ NCs.

The FTIR measurements provide more evidence that the final product is a combination of *h*-BN and *c*-Gd_2_O_3_ Ns. Figure 10 illustrates the results of FTIR studies to examine the chemical compositions and bonding properties of *h*-BNNs, *c*-Gd_2_O_3_Ns, and *h*-BN@Gd_2_O_3_NCs. The three samples' spectra have the same characteristics, including a strong absorption peak at 1631 cm^−1^ and a wide absorption band spanning 3000 cm^−1^ to 3700 cm^−1^^45^. Both of these absorptions were linked to the O–H vibration modes of bending and stretching, respectively. However, it was found that the three materials' IR absorption varied noticeably between 400 and 550 cm^−1^ (Fig. 10 inset). The Black spectrum reveals characteristic peaks at 416 cm^−1^, 455 cm^−1^, 490 cm^−1^, and 520 cm^−1^, attributed to the bending vibration of B–N and B–N–O, which are indicative of *h*-BNNs^34^. The red spectrum displays characteristic peaks at 404, 428, 447, 514, and 524 cm^−1^, attributed to the bending vibration of Gd-O and Gd-OH, characteristic of *c*-Gd_2_O_3_Ns^38,46^. Lastly, the blue spectrum exhibits characteristic peaks at 412 cm^−1^, 435 cm^−1^, 455 cm^−1^, 474 cm^−1^, 513 cm^−1^, and 520 cm^−1^, ascribed to the bending vibration of the triangular B–N–Gd, B–N–O, and Gd–O, which suggests the existence of *h*-BN@Gd_2_O_3_ NCs. The strength of other bands found in the FTIR spectrum is noticeably lower. The resulting product is a combination of *h*-BN and *c*-Gd_2_O3 Ns, as further evidenced by the FTIR data. The shifts in FTIR absorption bands in h-BNNs, c-Gd_2_O_3_Ns, and h-BN@Gd_2_O_3_NCs result from various factors, including the effect of the size. Smaller nanoparticles exhibit blue shifts in their FTIR bands due to quantum size effects. Additionally, differences in surface chemistry, confinement effects, aggregation, crystal structure, and chemical reactions contribute to band shifts. The surface chemistry of nanoparticles, their crystal structure variations, and chemical reactions with DDW can influence FTIR spectra.

**Figure 11:**
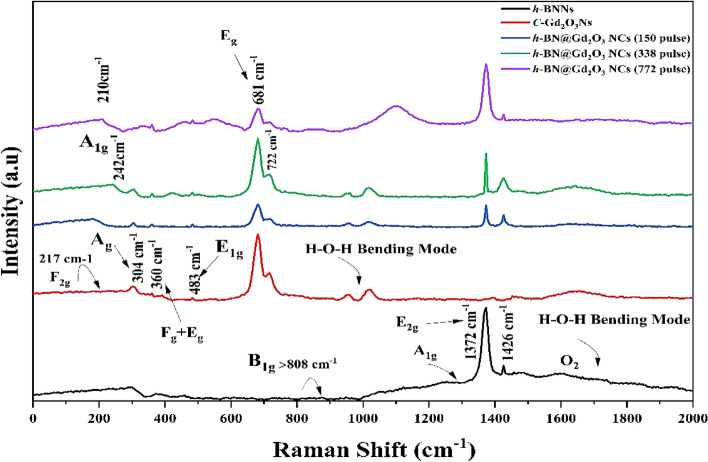
Raman spectrum of the prepared sample, *h*-BN Ns, *c*-Gd_2_O_3_ Ns, and *h*-BN@Gd_2_O_3_ NCs.

Raman spectra, the professional technique for material analysis, were utilized in this study to investigate various characteristics such as crystallographic phase, impurities, and structural defects. The Raman analyses employed a laser wavelength of (632.8 nm), and the obtained spectra ranged from 0 to 2000 cm^−1^ (Fig. 11). A comparison was made between the Raman spectra of *h*-BN@Gd_2_O_3_ prepared using different laser pulses, *h*-BNNs, and *c*-Gd_2_O_3_Ns prepared by the same technique. The Raman spectrum of the BNNs material exhibited two distinct bands at 1372, 1426, and a small peak in reign < 808 cm^−1^, corresponding to *h-*BN. The peak at < 808 cm^−1^ was attributed to the B1g phonon mode (which corresponds to the breathing mode of boron and nitrogen atoms in the hexagonal BN lattice). The peak at 1372 cm^−1^ was attributed to the E_2g_ phonon mode (it corresponds to the in-plane vibration of boron and nitrogen atoms in a hexagonal lattice), similar to the G peak observed in graphene. The peak at 1426 cm^−1^ was attributed to the A_1g_ phonon mode (it is related to the out-of-plane vibrations of boron and nitrogen atoms in a hexagonal BN structure)^35,47, 48^. In contrast, the Raman spectra of Gd_2_O_3_ revealed five prominent peaks at approximately 210, 304, 360, 483, 681, and 722 cm^–1^, which corresponded to F_2g_ (This mode is associated with oxygen vibrations in the lattice), A_g_ translatory (It is attributed to stretching vibrations of oxygen atoms), F_g_ + A_g_, and E_1g_ liberation modes^49,50^, showing the existence of c-Gd_2_O_3_Ns for the doped sample's h-BN phase, c-Gd_2_O_3_ at the location of nine significant Raman peaks is seen at 210 cm^−1^, 242 cm^−1^, 304 cm^−1^, 360 cm^−1^, 483 cm^−1^, 681 cm^−1^, 722 cm^−1^, 1372 cm^−1^, and 1426 cm^−1^. These findings suggest that the nanostructured *h*-BN@Gd_2_O_3_ samples consist of a mixture of *h*-BN and *c*-Gd_2_O_3_ nanostructures.

The wave numbers of the corresponding Raman peaks for the hexagonal^47^ and cubic^51^ phases of doped and undoped *h*-BN and *c*-Gd_2_O_3_ are listed in Table 2. Notably, the intensity of the Raman bands in the spectra increased with the number of pulses, indicating a correlation between the concentration of the studied molecules and the intensity of the Raman spectral bands. The observed Raman bands align with those reported by other researchers^48,52–55^. Additionally, this study represents the first Raman analysis conducted on the *h*-BN@Gd_2_O_3_ cubic phase. Furthermore, the Raman spectra of *h*-BN exhibited two broad bands around 1600 cm^−1^, associated with O_2_ and H–O–H bending modes, while Gd_2_O_3_'s Raman spectra showed a wide band at 1000 cm^−1^, corresponding to the H–O–H bending mode, both originating from the liquid environment. The shift of Raman modes can occur due to various factors, including the presence of oxygen vacancies, defects induced by disorder, or the effects of phonon confinement^56^.

**Table 2:** Data obtained via the Raman spectra of *h*-BN Ns, *C*-Gd_2_O_3_Ns, *h*-BN@Gd_2_O_3_ NCs (150, 338, and 772 pulses) prepared by LEFL in DDW at room temperature.

Raman band location (cm^−1^) from BN of the hexagonal type (Ref. ^47^)	Raman band location (cm^−1^) from Gd_2_O_3_ of the cubic type (Ref. ^51^)	Raman band location (cm^−1^) from BNNs of the hexagonal type (this work)	Raman band location (cm^−1^) from the Gd_2_O_3_Ns of the cubic type (this work)	Raman band location (cm^−1^) from three samples of *h*-BN@Gd_2_O_3_ NCs (this work)
808 (B_1g_)	81 m	1372	–	1372
1365 (E_2g_)	85 vw	1426	–	1426
1370	95 s	–		–
1375 (A_1g_)	108 vw	–		–
1450	119 s	–	–	–
–	135 w	–	–	–
–	145 w	–	–	–
–	198 w	–	–	–
–	235 w	–	217	210
–	299 vw	–	–	260
–	316 m	–	304	304
–	337 vw	–	348	348
–	361 vs	–	360	360
–	401 w	–	–	–
–	413 vw	–	–	–
–	435 sh	–	–	–
–	447 m	–	–	458
–	479 vw	–	483	483
–	568 m	–	–	–
–		–	549	549
–		–	681	681
–			722	722

*vw* very weak, *w* weak, *m* medium, *s* strong, *vs* very strong, *sh* shoulder.

**Figure 12:**
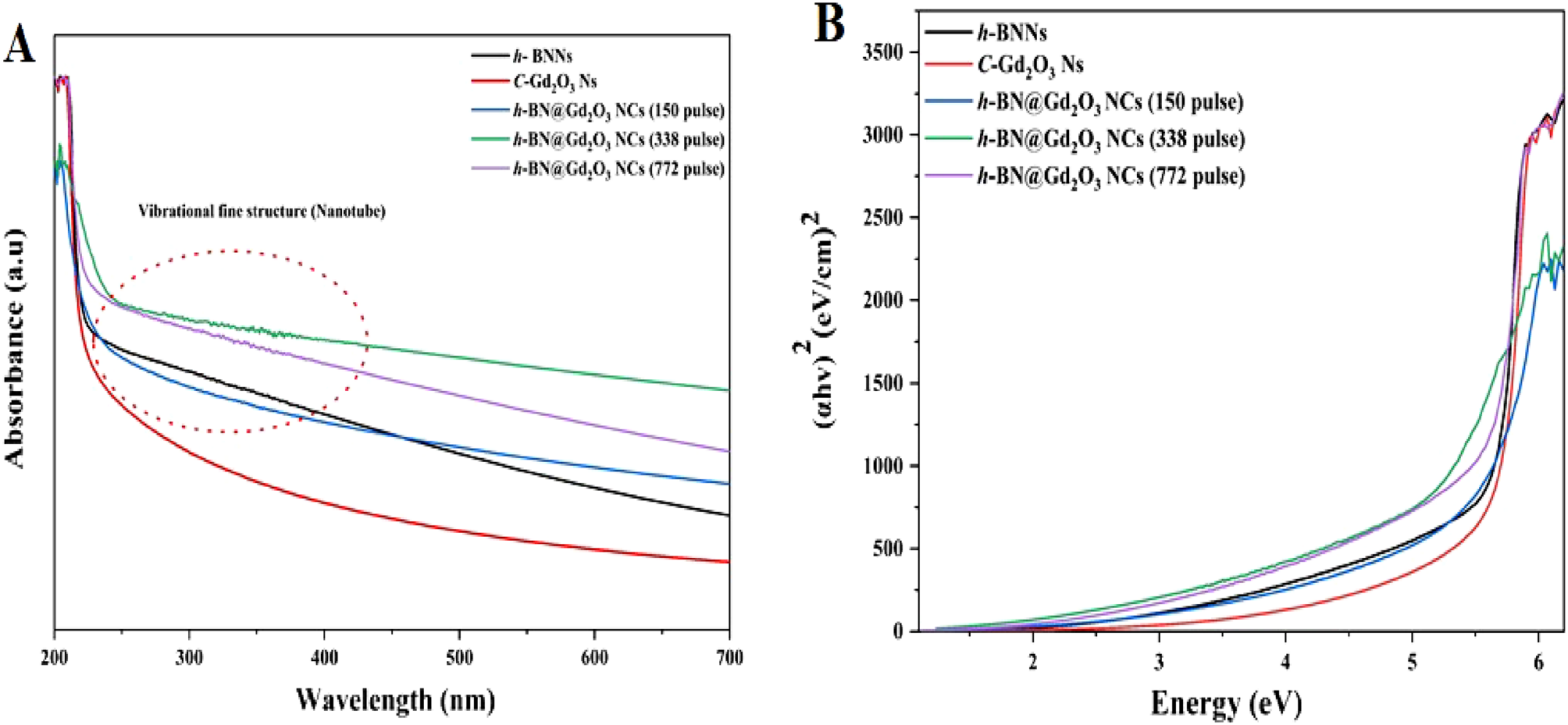
(**A**) UV–VIS spectrums, and (**B**) band gap of the prepared samples, *h*-BN Ns, *c*-Gd_2_O_3_ Ns, and *h*-BN@Gd_2_O_3_ NCs with different laser pulses (150, 338, and 772).

Figure 12A illustrates the UV–vis intensity of *h*-BNNs, *c*-Gd_2_O_3_Ns, and *h*-BN@Gd_2_O_3_NCs, synthesized using a laser energy of 10 mJ with varying numbers of laser pulses (150, 338, and 772). The recorded spectra show a progressive enhancement in absorption intensity between 200 and 250 nm as the number of laser pulses increases.^57,58^ This trend is likely attributed to all samples' heightened concentration and reduced size of nanoparticles. The UV spectra of *h*-BNNs and NCs samples reveal an absorption peak below 380 nm, due to fine structural vibrations and band-to-band electron transitions (as depicted in Fig. 12A). Moreover, a smooth decline is observed at longer wavelengths. The higher energy of laser pulses causes target evaporation, melting, and phase transformation at elevated temperatures and pressures, accompanied by an extended irradiation time. Several parameters, including stoichiometry, morphologies, and size distribution, influence the absorption behavior of the NCs^59^. Importantly, the absorption curves below 380 nm exhibit an increase, indicating a hypsochromic shift (blue shift) in the absorption values with an increasing number of laser pulses. The quantum confinement effect can be credited with this change. Oxygen deficiency and lattice strain resulting from the ablation of Gd_2_O_3_^23,60–62^.

Figure 12B illustrates the optical band gap of the samples, offering a window for the PL spectrum of the nanoparticles (NPs). The impact of nanocrystal size on the electronic structure of semiconducting NPs is highlighted by the rise in band gap energy coupled with the decrease in particle size. This phenomenon is widely recognized as the quantum confinement effect, and its assessment was conducted using the Tauc method. The Tauc method postulates that the energy-dependent absorption coefficient α can be expressed by Eq. (4)^63,64^:

4 $$\left(\alpha .hv\right){1}/{\gamma }= \beta \left(hv-Eg\right).$$

Here, the symbol h represents the Planck constant, v indicates the frequency of the photon, Eg represents the band gap energy, and β is a constant. The γ factor varies based on the nature of the electron transition and takes values of 1/2 or 2 for direct and indirect transition band gaps, respectively^60^. Band gap energies of 5.6, 5.7, 5.6, 4.9, and 5.5 eV were recorded for different materials, including *h*-BN Ns, *c*-Gd_2_O_3_Ns, and three unique *h*-BN@Gd_2_O_3_ NC samples produced using varying laser pulse numbers (150, 338, and 772). The decrease in band gap energy in the doped samples confirms the effective incorporation of *c*-Gd_2_O_3_Ns into the *h*-BN structure, highlighting their potential in materials science applications.

**Figure 13:**
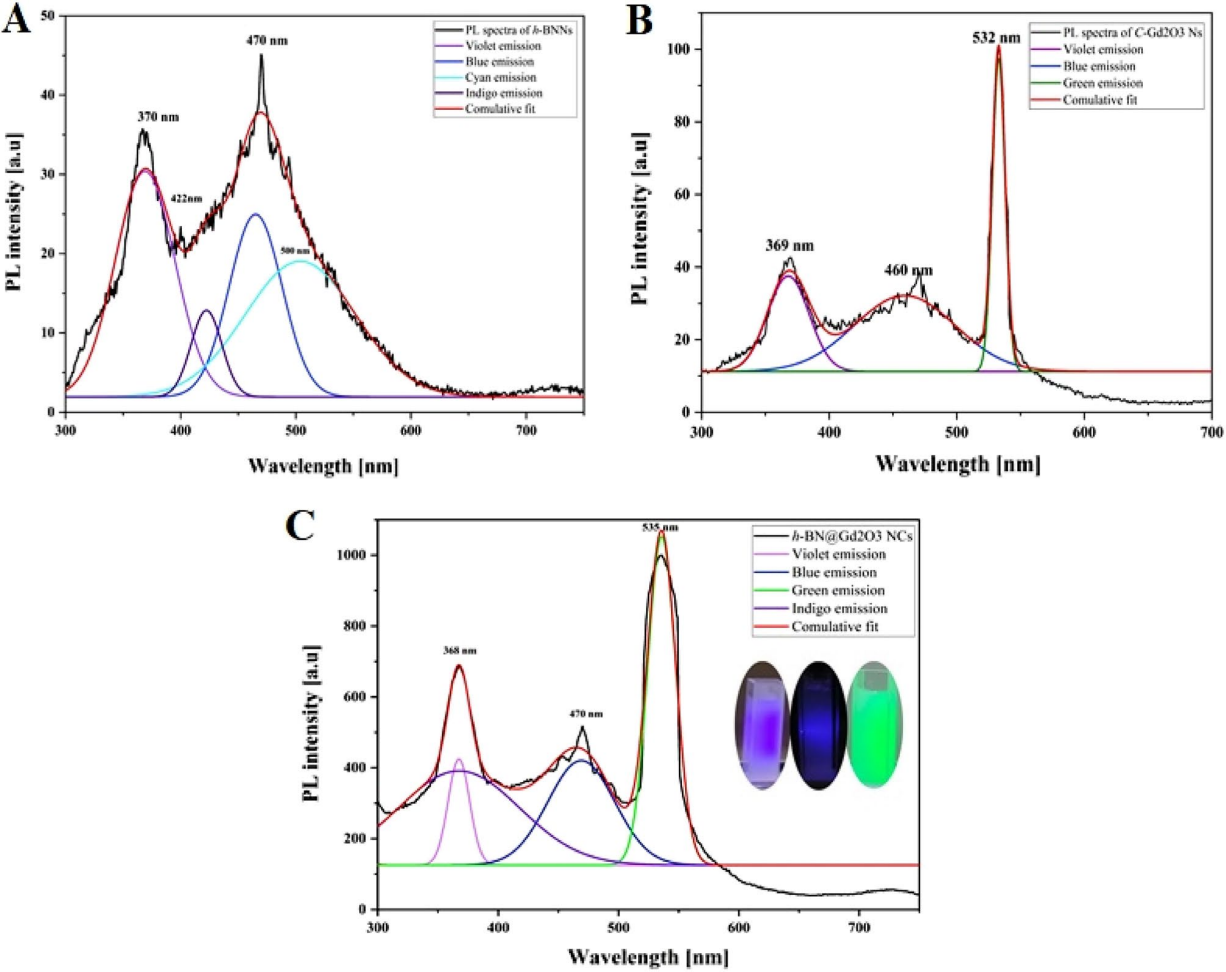
Deconvolution of fluorescence spectrum for the samples: (**A**) *h*-BNNs, (**B**) *c*-Gd_2_O_3_Ns, and (**C**) *h*-BN@Gd_2_O_3_NCs (338 pulses).

Figure 13A–C shows the spectrum using a 532 nm excitation wavelength and deconvolution peaks of PL emission for three different samples: *h*-BNNs, *c*-Gd_2_O_3_Ns, and *h*-BN@Gd_2_O_3_ NCs (338 pulses) in DDW. The deconvolution process utilized multi-Gaussian functions to obtain these contributions. In Fig. 13, the PL spectra underwent deconvolution, specifically focusing on the PL peaks, using Origin Lab software, while the other components were fitted with a Gaussian line shape. Following the deconvolution, we assigned labels to the peaks based on their wavelength and color-coded each peak according to its emission band for each sample. Our findings indicate that the PL spectra of *h*-BNNs exhibit four distinguishable spectral features in the blue range (~ 300 to 550 nm): Violet (370 nm, 3.35 eV), Indigo (422 nm, 2.9 eV), Blue (470 nm, 2.64 eV), and Cyan (500 nm, 2.48 eV) emission. For *c*-Gd_2_O_3_Ns, three distinguishable spectral features were observed in the UV-blue-green range (~ 300 to 500 nm): Violet (369 nm, 3.36 eV), Blue (460 nm, 2.7 eV), and Green (532 nm, 2.3 eV) emission. However, for *h*-BN@Gd_2_O_3_NCs (338 pulses), three spectral features were detected in the UV-blue-green range (~ 300 to 550 nm): Violet (368 nm, 3.36 eV), Blue (470 nm, 2.64 eV), and Green (535 nm, 2.3 eV) emission. Figure 13C represents the ultraviolet, blue, and green regions, the green emission peak corresponds to the single ionized oxygen vacancy^65^. It is evident from Fig. 13A that the Green bands are absent in the spectrum of *h*-BNNs, suggesting their origin from the *c*-Gd_2_O_3_ Ns. The Blue band can be attributed to traces of *h*-BNNs emission originating from the NCs matrix. We observe that the intensities and spectral positions of the bands change with variations in the concentration of NPs, resulting in an increase in the PL of *h*-BN@Gd_2_O_3_ NCs^29^. The fitting analysis of Fig. 13C reveals that as the concentrations of *h*-BNNs and *c*-Gd_2_O_3_Ns increase, the intensity of the UV, Blue, and Green bands also increases. Therefore, the fitting results demonstrate that the PL spectra can be described by four PL components, which exhibit different trends with changes in NPs. The above results indicate the successful synthesis of *h*-BNNs, *c*-Gd_2_O_3_Ns, and h-BN@Gd_2_O_3_ NCs, all of which exhibit stable and strong fluorescence in water. The introduction of Gd_2_O_3_ to BN nanoparticles can elicit various effects on PL spectra. These effects depend on factors such as the concentration and specific properties of the Gd_2_O_3_ and BN NPs. One notable effect is the PL intensity enhancement, where Gd_2_O_3_ nanoparticles, under certain conditions, act as sensitizers, boosting the PL intensity of BN nanoparticles. This phenomenon is often observed in rare-earth-doped materials, where Gd_2_O_3_ transfers energy to BN, resulting in heightened luminescence (Fig. 13C). Another effect is the emission wavelength shift, where the inclusion of Gd_2_O_3_ induces a shift in the emission wavelength of BN nanoparticles. This shift can manifest as a movement toward longer or shorter wavelengths, contingent on the specific interactions between these materials. The doping-related bands have a significant effect, as Gd_2_O_3_ introduction can result in new PL bands associated with gadolinium emissions. These bands may intersect or interact with the PL bands of BN, causing changes in the PL spectrum. Gd_2_O_3_'s capacity for energy transfer is also noteworthy, influencing the excitation and relaxation dynamics of BN nanoparticles. This can lead to alterations in PL kinetics and spectral characteristics. Surface effects are yet another factor to consider, as the characteristics of the surface and interactions at the Gd_2_O_3_-BN interface can influence PL spectra. Surface states or introduced energy levels due to the presence of the Gd_2_O_3_ layer can affect PL characteristics. The specific effects observed depend on variables like Gd_2_O_3_ concentration, nanoparticle size, structure, and synthesis conditions. Understanding these effects is crucial when exploring the potential applications of Gd_2_O3-enhanced BN nanoparticles in various fields, from optoelectronics to biomedicine.

**Figure 14:**
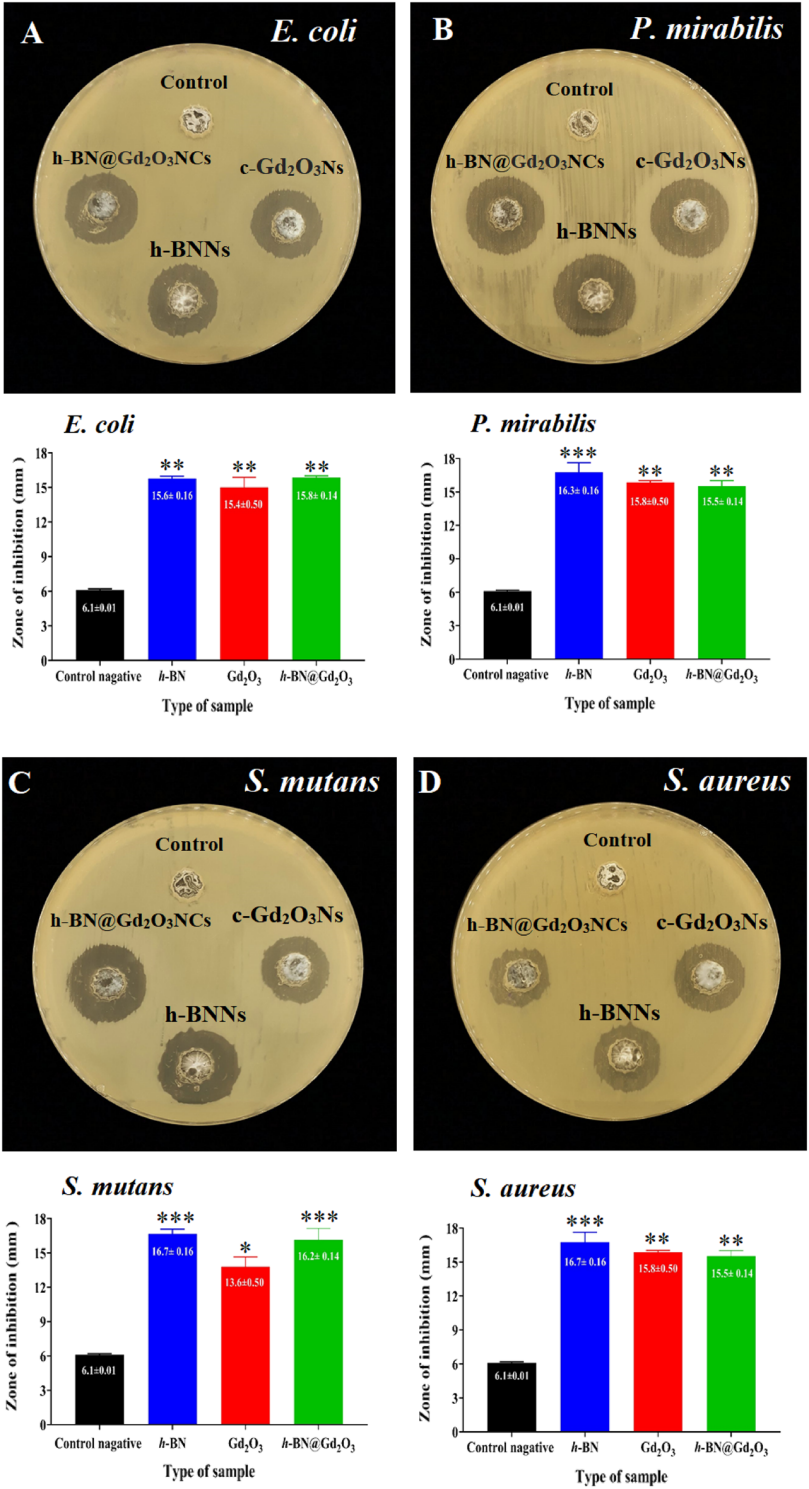
Antibacterial activity of negative control (DDW), *h*-BNNs, *c*-Gd_2_O_3_Ns, and *h*-BN@Gd_2_O_3_ NCs. Zone of inhibition and graphical representation of (**A**) *E. coli*, (**B**) *P. mirabilis*, (**C**) *S. mutans*, and (**D**) *S. aureus*. *p ≤ 0.05, ** p ≤ 0.01, *** p ≤ 0.001.

The well diffusion method has been used to investigate the antibacterial activity of *h*-BNNs, *c*-Gd_2_O_3_Ns, and *h*-BN@Gd_2_O_3_ NCs (338 pulses) against *S. aureus and S. mutans* and *E. coli* and *P. mirabilis*. The inhibition zones of the prepared colloidal Ns are listed in the graphical representation of all measured zones of inhibition after 24 h of incubation period at 37C^˚^. The results show that the prepared nanostructures were more effective in the growth of Gram-ve bacteria than Gram + ve bacteria. For *h*-BNNs, the highest activity was recorded against *P. mirabilis* with the inhibition zone reaching 16.3 mm, followed by *E. coli* which exhibited the highest diameter of inhibition zone that reached 15.8 mm after treatment with h-BN@Gd_2_O_3_ NCs, and the lowest diameter of the inhibition zone was 15.4 mm after treatment with *c*-Gd_2_O_3_Ns. For Gram + ve bacteria, the highest effect for h-BNNs and *h*-BN@Gd_2_O_3_ NCs) was in *S. mutans* with an inhibition zone diameter reached 16.7 and 16.2 mm, and the lowest inhibition zone diameter was 13.6 mm for the *c*-Gd_2_O_3_Ns. The inhibition zone of *S. aureus* was 15.2 mm for *h*-BNNs, followed by (*c*-Gd_2_O_3_Ns) in diameter reached 15 mm, and the lowest inhibition zone diameter was 13 mm for *h*-BN@Gd_2_O_3_ NCs (Fig. 14).

Table 3 presents a comparison between our synthesized nanocomposite and previously reported antibacterial nanomaterials. The results in Table 3 indicate that our prepared h-BNNs, c-Gd_2_O_3_Ns, and h-BN@Gd_2_O_3_ NCs nanocomposite were more potent than recorded previously. Therefore, it can be concluded that h-BNNs, c-Gd_2_O_3_Ns, and h-BN@Gd_2_O_3_ NCs have the potential to serve as next-generation antibacterial agents for a range of biomedical applications.

**Table 3:** Comparison of previously reported antibacterial nanomaterials and our purposed h-BNNs, c-Gd_2_O_3_Ns, and h-BN@Gd_2_O_3_ NCs.

Materials	Zone of inhibition (mm)				References
	*E. coli*	*P. mirabilis*	*S. mutans*	*S. aureus*	
BN nanosheet	–	–	11.0	–	^66^
BN nanosheet	0.45	–	–	0.35	^67^
Gd_2_O_3_-MWCNT	23.0	–	–	14.0	^68^
Bi@BN	3.65			4.35	^67^
Zr-doped BN	5.00	–	–	4.00	^69^
Cu-doped BN nanosheets	3.65	–	–	4.25	^70^
h-BNNs	15.6	16.3	16.7	15.2	This study
c-Gd_2_O_3_Ns	15.4	15.8	13.6	15.0	This study
h-BN@Gd_2_O_3_	15.8	15.5	16.2	13.0	This study

**Figure 15:**
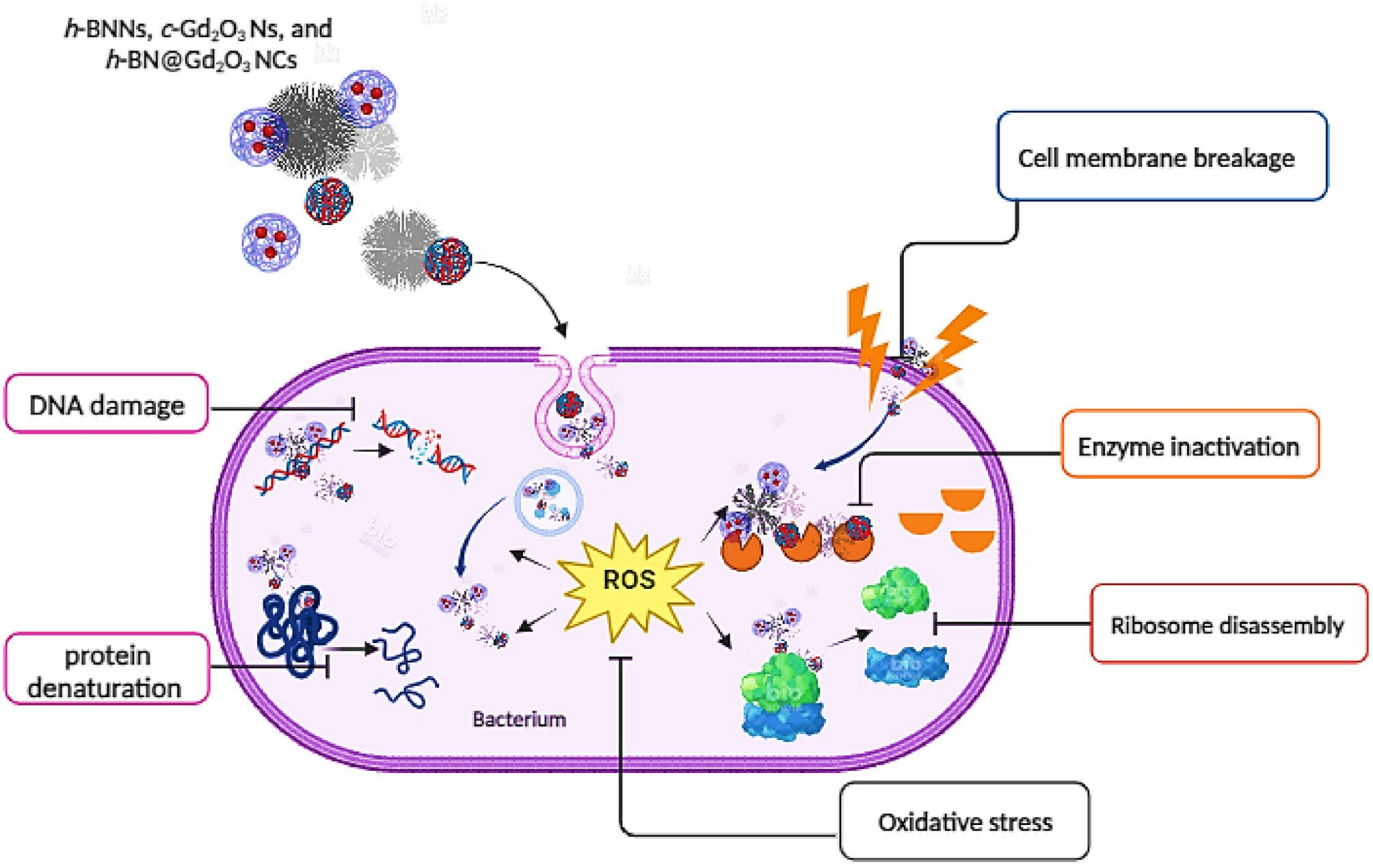
The mechanism of antibacterial activity of *h*-BNNs, *c*-Gd_2_O_3_Ns, and *h*-BN@Gd_2_O_3_ NCs.

The antibacterial efficacy of the synthesized Ns can be attributed to their interaction with cell wall constituents, causing structural changes potentially affecting cell membranes. These findings stem from the close contact between the *c*-Gd_2_O_3_Ns (shell) and the *h*-BNNs (core), facilitating direct engagement with the membrane and ultimately leading to the rupture of contracted bacteria. The escalating integration of nanostructures in medicine has sparked numerous investigations into their potential antibacterial mechanisms. Metal nitride and oxides, for instance, can modulate bacterial metabolic activity, offering a promising strategy for disease treatment. The effective antibacterial action of nanostructures hinges on their direct interaction with bacterial cells. This interaction is achieved through mechanisms such as electrostatic attraction^71^, the forces of van der Waals^72^, the interactions between receptors and ligands^73^, and hydrophobic bonds^74^. Subsequently, nanoparticles (NPs) traverse bacterial membranes and accumulate along metabolic pathways, influencing cellular membrane structure and function. NPs also engage with fundamental components of bacterial cells, including DNA, where they induce disruptions through various means, including endogenous and exogenous damage, lysosomes, ribosomes, and enzymes, among others^75^. This interaction causes oxidative stress, as endogenous sources trigger the formation of ROS during normal cellular metabolism^76^. These highly unstable free radicals can instantaneously react with other substances. The interaction of free radicals with DNA initiates a cascade of events resulting in genotoxic lesions. This process initiates diverse changes, including heterogeneous alterations, shifts in cell membrane permeability, and disruptions in electrolyte balance, enzyme inhibition, protein deactivation, and modifications in gene expression^77–80^. Notably, contemporary research has proposed several prevalent mechanisms, which include oxidative stress^81^, the release of metal ions^82^, and non-oxidative pathways^83^. The various mechanisms through which nanoparticles combat bacteria are explained in Fig. 15.

**Figure 16:**
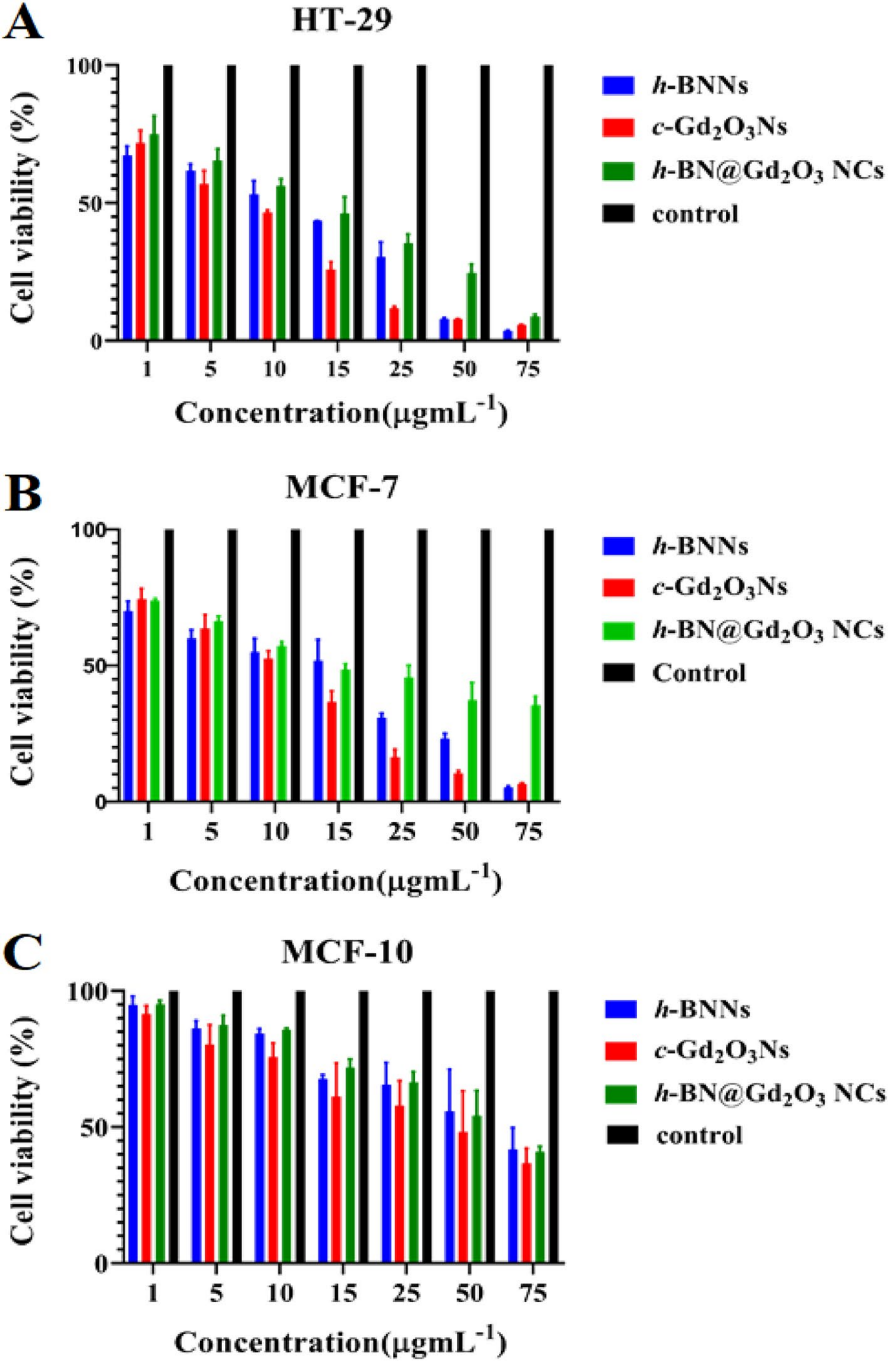
Cytotoxicity effects of (h-BNNs, c-Gd_2_O_3_Ns, and *h*-BN@Gd_2_O_3_ NCs) treated with (**A**) HT-29, (**B**) MCF-7, and (**C**) MCF-10 cell lines. The data were collected over a 24-h treatment, and the values reflect the mean standard deviation of three experiments.

The cellular toxicity of *h*-BNNs, *c*-Gd_2_O_3_Ns, and *h*-BN@Gd_2_O_3_ NCs was performed to evaluate the viability of MCF-7, MCF-10, and HT-29 cell lines using the MTT assay. Different concentrations (1, 5, 10, 15, 25, 50, and 75 µg/mL) of *h*-BNNs, *c*-Gd_2_O_3_Ns, and *h*-BN@Gd_2_O_3_ NCs were used and incubated for 24 h. As the concentration of nanomaterials increases, the violet color of the MTT assay becomes progressively lighter. This color change indicates a decrease in the number of viable cells. The efficacy of nanomaterials against biological systems and cells holds considerable importance for fulfilling biomaterial criteria, rendering them suitable for medical applications. The outcomes of the MTT assay are illustrated in Fig. 16A–C.

The findings illustrated that treatment with *h*-BNNs, *c*-Gd_2_O_3_Ns, and *h*-BN@Gd_2_O_3_ NCs inhibited the growth of cells significantly (P < 0.05) as compared to those of control cultures, and the reduction was concentration-dependent. The highest inhibition was found in the HT-29 cells, followed by MCF-7 cells, with less cytotoxicity against MCF-10. For HT-29, the inhibitory concentration value (IC_50_) was 12 µg mL^−1^, 10 µg mL^−1^, and 15 µg mL^−1^, respectively. While IC_50_ for MCF-7 cells reveals 14 µg mL^−1^, 15 µg mL^−1^, and 28 µg mL^−1^, respectively. The Ns used in this investigation had particles that were 30 nm in size. The outcomes from XRD and the results of PL analysis showed that there are multiple interstitial oxygen vacancies (O_i_) at 532 nm linked to the sample's oxygen vacancies (O_v_). These alterations are attributed to the substantial quantity of ROS generated across the three samples. These defects allow a greater number of electron–hole pairs to travel toward the nanomaterial matrix's surface within the specimens. Additionally, the samples' aqueous environment (DDW) contains singlet oxygen (^1^O_2_), hydroxyl radicals (OH), and superoxide anions (O_2_), which the electrons and holes interact with and may cause to produce ROS. These free radicals can trigger oxidation and reduction reactions in macromolecules, including proteins, lipids, and nucleic acids, which leads to oxidative stress when they come into contact with the cellular environment. As a result, a state of disequilibrium develops inside the cells as a result of the buildup of ROS, outpacing the biological system's ability to quickly remove these reactive radicals or repair the harm done by tumor cells^84–86^.

In the context of *h*-BNNs and *c*-Gd_2_O_3_Ns, MTT testing has been conducted to assess their cytotoxicity in MCF-10 cells. The results indicate that both *h*-BNNs and *c*-Gd_2_O_3_Ns demonstrate minimal cytotoxicity and are deemed compatible with MCF-10 cells, aligning with previously published studies^87–91^. However, uncertainties persist regarding the toxicity of *h*-BNNs and *c*-Gd_2_O_3_Ns, and their full biocompatibility remains unproven^4,92^. These findings suggest the potential utility of these materials in biomedical applications requiring interaction with living tissues. Nevertheless, further research is imperative to comprehensively comprehend their behavior in various biological systems and ensure their safety for medical applications.

Previous studies revealed that the cell viability of cancer and normal cells after treatment with boron nitride decreased as a function of concentration and time. Cancer cell lines MCF-7 and Hela showed 45% and 60% cytotoxicity, respectively at a concentration of 2 mg/mL for 24 h treatment, which further increased up to 60% and 70%, respectively in 48 h treatment. Whereas in normal cell line (HEK-293) 30% cytotoxicity was observed at a higher concentration (2 mg mL^−1^) and 24 h of treatment, which increased up to 50% after 48 h. It is evident that the cytotoxicity of BN nanostructures is higher in cancer cells as compared with normal cell lines^93^.

**Figure 17:**
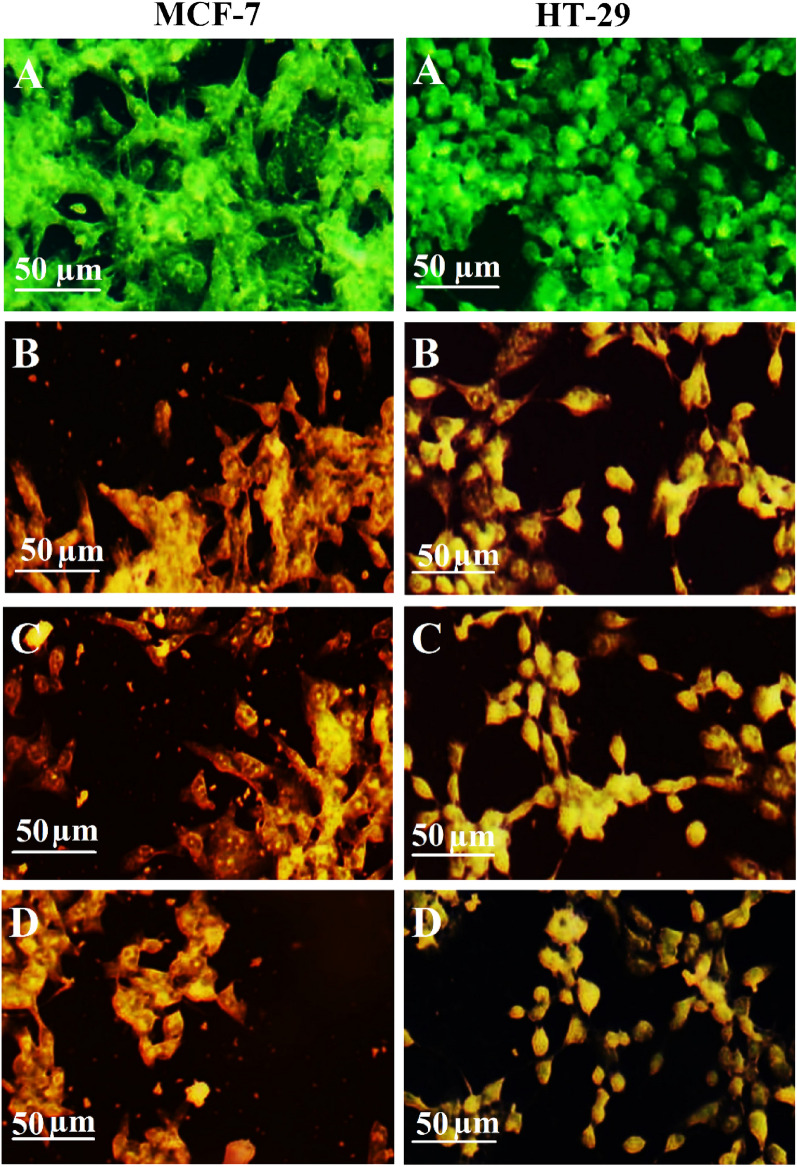
MCF-7 cells and HT-29 cells with dual-dye AO/EB staining. (**A**) Non-treated cells (Control) and cells treated with IC_50_ of (**B**) h-BNNs, (**C**) c-Gd_2_O_3_Ns, and (**D**) h-BN@Gd_2_O_3_ NCs.

Cytotoxicity associated with *h*-BNNs, *c*-Gd_2_O_3_Ns, and *h*-BN@Gd_2_O_3_ NCs involves apoptotic changes and nuclear condensation. To identify and quantify the induction of apoptosis and the formation of necrosis in HT-29 and MCF-7 cells following exposure to *h*-BNNs, *c*-Gd_2_O_3_Ns, and *h*-BN@Gd_2_O_3_ NCs, fluorescent AO/EB DNA-binding dyes were utilized. The AO dye was absorbed by viable cells, as indicated by green fluorescence, while the EB dye exclusively interacted with deceased cells, resulting in red fluorescence. Within viable cells (non-treated cells), distinct bright uniform normal green nuclei with an organized structure. While treated cells were significantly enhanced and distinguished according to the fluorescence emission the apoptotic cells have yellow nuclei. (depicted in Fig. 17A–D. The anticancer activity ascribed to BN, Gd, and O_v_ in h-BN, *c*-Gd_2_O_3_, and *h*-BN@Gd_2_O_3_ NCs, corresponds with increased ROS production. MTT assays performed on HT-29 and MCF-7 cells revealed the potential anticancer effects of *h*-BNNs, *c*-Gd_2_O_3_Ns, and *h*-BN@Gd_2_O_3_NCs. Moreover, the demise of cancer cells induced by nanoparticles occurred through apoptosis, as visualized by AO/EtBr staining.

One proposed mechanism of the anticancer activity of these nanoparticles can be attributed to the ability of NPs to produce ROS, which can cause changes in biomacromolecules, such as proteins, nucleic acids, and lipids in response to the effects of the generated oxidative stress in the cells and tissues. ROS produce free radicals that are short-lived and unstable, which influence the nuclear viability and healthiness conditions of the affected organisms, and finally lead to cell death. ROS also causes oxidation of proteins and peroxidation of lipids that leads to damaging the fluidity of the cell membrane, thereby changing the permeability of fluids and ion transports across it, and causing inhibition of metabolic processes^31,32^.

Finally, *h*-BNNs, *c*-Gd_2_O_3_Ns, and *h*-BN@Gd_2_O_3_NCs emerge as highly promising materials for cancer therapy due to their notable attributes, including strong chemical stability, uniformity, and excellent dispersibility in solution. Unique structural features, such as the spherical nanosheets of BN and the needle-like surface of *c*-Gd_2_O_3_Ns, position *h*-BNNs and *c*-Gd_2_O_3_ as ideal nanocarriers for targeted delivery of anticancer drugs, potentially enhancing their penetration into endosomes. Future research efforts can be directed towards further functionalizing *h*-BNNs to enhance their biological performance, similar to BNNTs.

Previous study has elucidated diverse responses of cancer cell lines to treatments involving boron nitride (BN) and gadolinium oxide (Gd_2_O_3_). BN has demonstrated effectiveness against neoplastically transformed IAR-6-1 cells^87^, breast cancer cells (4T1)^89^, prostate cancer cells (DU145 and PC3)^94^, as well as BV2 mouse glioma cells, GMI-R1 rat microglial cells, and human cervical carcinoma cells (HeLa), although its efficacy in other cancer types remains relatively unexplored^95^. The cytotoxicity of h-BNNDs against HUVEC cells (human umbilical vein endothelial cells) was also investigated by Mao et al.^96^. They found that the assessment of cell viability combined with cell counting, viability/cytotoxicity assays, and cell apoptosis detection demonstrated that BNNDs could not elicit significant acute cytotoxicity. They proved that the cell viability remained higher than 80% even at high concentrations and long incubation (200 μg mL^−1^, 48 h).

On the other hand, Gd_2_O_3_ nanoparticles have exhibited promise in treating M109 (malignant tumors of the mouse pulmonary system), 4T1 (epithelial breast carcinoma) cell lines^88^, mouse colon adenocarcinoma CT26 cell line^97^, HeLa, and COC1/DDP cells in the context of ovarian cancers^98^. Previous results on the viability of the Ba/F3 cells (a murine interleukin-3 dependent pro-B cell line) and THP-1 cells (human monocytic cell line) showed that the cells were viable after incubation with Gd_2_O_3_ nanoparticles and remained intact at the time of MRI measurement^99^.

The in vitro cytotoxicity test on normal (HaCaT) and cancerous (HeLa) cells reveals that the CNT/Gd_2_O_3_ hybrid nanostructure is bio-compatible in comparison to the pure CNTs^99^.

However, this study underscores the potential of BN, Gd_2_O_3_, and their combination in cancer treatment. Nevertheless, further research is imperative to elucidate the underlying mechanisms and optimize their therapeutic applications across wide spectrum of cancer cell lines. However, toxicology investigations using in vivo studies of prepared nanoparticles need more thorough viability studies, and long-term biological effects need to be evaluated.

**Figure 18:**
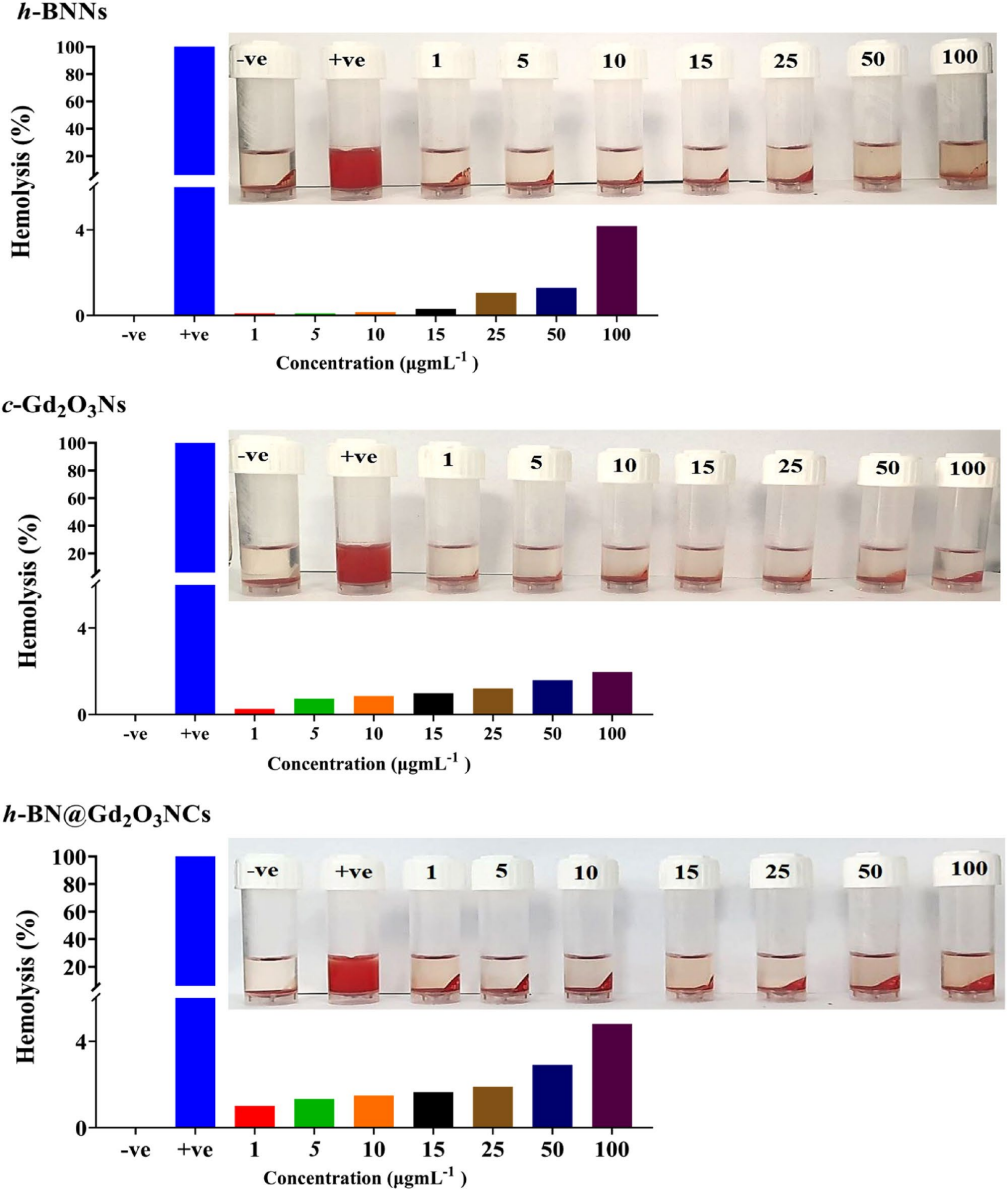
Hemolysis evaluation of erythrocytes subjected to incubation with deionized water, PBS, h-BNNs, c-Gd_2_O_3_Ns, and h-BN@Gd2O3 NCs, with accompanying histogram data.

Additionally, an assessment of the hemolytic effect using an ex vivo incubation with erythrocytes revealed that hexagonal boron nitride (h-BN) nanosheets, cubic gadolinium oxide (c-Gd_2_O_3_) nanoparticles, and h-BN@Gd_2_O_3_ nanocomposites demonstrated minimal hemolysis rates, registering at less than 5%, even when exposed to concentrations up to 100 μg mL^−1^ (Fig. 18). The data is consistent with a previous study that demonstrated Gd_2_O_3_@PCD-FA showed no cytotoxic effect on MCF10A cells (normal human breast cells) and exhibited acceptable hemocompatibility against human RBCs, revealing their biocompatible properties^100^. Another study also revealed that BN has no significant hemolysis rate on human RBCs even when the concentration reached 100 μg mL^−1^^90^. The hemolysis assay serves as a preliminary analytical approach for ascertaining the compatibility of nanoparticles (NPs) with blood and for assessing their effects on erythrocyte well-being. These observations underscore the potential of h-BN@Gd_2_O_3_ as a viable candidate for drug delivery and demonstrate its good cytocompatibility for biomedical applications.

## Conclusion

In summary, a novel, cost-effective hybrid method was utilized to synthesize new nanomaterials, namely *h*-BNNs, *c*-Gd_2_O_3_Ns, and *h*-BN@Gd_2_O_3_ NCs. The LEFL process involved the use of Nd: YAG lasers with varying wavelengths (1064 and 532 nm) and pulse numbers (150, 338, and 772). The resulting *h*-BNNs displayed small particles with irregular spherical shapes and nanosheets, while Gd_2_O_3_Ns exhibited nanofiber-like, needle-like, and sheet-like structures (leaf-like). TEM analysis corroborated the findings from FESEM images. Phase analysis unveiled hexagonal and cubic structures, with average crystallite sizes of 7.04 nm for *h*-BNNs, 0.68 nm for *c*-Gd_2_O_3_Ns, and 3.75 nm for *h*-BN@Gd_2_O_3_ NCs. These findings collectively endorse the credibility of HRTEM and SAED methodologies in characterizing nanoscale crystal structures. Notably, these results harmonize seamlessly with the XRD findings discussed earlier, solidifying their reliability. Raman spectroscopy and FTIR spectroscopy provide complementary insights into the structure and composition of materials. Raman spectroscopy detects homo-nuclear molecular bonds and reveals details about both intra- and intermolecular vibrations. On the other hand, FTIR spectroscopy is attuned to hetero-nuclear functional group vibrations and polar bonds, thereby enhancing the understanding of material properties. All samples showed absorption band edge values between 200 and 250 nm. The PL spectrum highlighted the presence of numerous O_v_ that brought on cell death and the creation of ROS. The samples exhibited higher antibacterial activity against both bacterial strains. Furthermore, all three samples demonstrated substantial anticancer effects against HT-29 and MCF-7 cells. Moving forward, we anticipate that *h*-BNNs, *c*-Gd_2_O_3_Ns, and *h*-BN@Gd_2_O_3_ NCs could potentially serve as promising anticancer agents and biocompatible, with further in vivo experiments worthy of exploring their antitumor effect.

## Data Availability

All data are included in this published article.
